# Social determinants of mental health in Germany: a systematic scoping review mapping the landscape of researched determinants, outcome measures, and explanatory concepts

**DOI:** 10.1186/s12939-026-02877-0

**Published:** 2026-06-04

**Authors:** Martin S. Lehe, Pauline Reiß, Vanessa C. Jürgensen, Georg Halbeisen, Georgios Paslakis

**Affiliations:** 1 Medical Faculty, Campus East-Westphalia, University Clinic for Psychosomatic Medicine and Psychotherapy, Ruhr University Bochum, Virchowstr. 65, 32312 Lübbecke, Germany; 2 Institute for Diversity Medicine, Ruhr-University Bochum, Bochum, Germany; 3 Department of Clinical Psychology and Psychotherapy, University of Bamberg, Bamberg, Germany; 4 Department of Business Administration and Economics, OWL University of Applied Sciences and Arts, Lemgo, Germany

**Keywords:** Social determinants, Social determinants of health, Mental health, Intersectionality, Health care inequities, Gender, Systematic review

## Abstract

**Introduction:**

Social determinants play a critical role in shaping mental health (MH) outcomes. The World Health Organization emphasizes the importance of addressing such factors to reduce MH disparities. This scoping review aims to assess the landscape of research on social determinants of MH in Germany, focusing on frequently studied social determinants, MH outcomes, and underlying theoretical frameworks and explanatory mechanisms, particularly the consideration of the framework of intersectionality.

**Methods:**

Following the Preferred Reporting Items for Systematic reviews and Meta-Analyses extension for Scoping Reviews guidelines, a systematic literature search was conducted in PubMed and Web of Science. Studies that specifically addressed social determinants of MH in Germany were targeted, and data were charted to map key research trends.

**Results:**

A total of 73 studies were included in the data analysis and synthesis, and outcomes were grouped into eight social determinants of health domains. The most frequently examined domains were demographics (in 96.0% of included studies), interpersonal/community/cultural influences (89.3%), economic stability (72.0%), and education (57.3%). Less frequently addressed domains included neighborhood and built environment (25.3%), environmental events (16.0%), other health-related determinants (16.0%), and healthcare access and quality (5.3%). We observed substantial heterogeneity within and between domains. MH outcomes were more often studied in terms of mental illness than positively defined MH, with depression and anxiety being the most frequently assessed outcomes. Theoretical frameworks such as the biopsychosocial model, social-ecological perspectives, and resilience theory were commonly applied, while intersectionality was rarely explicitly analyzed.

**Discussion:**

This scoping review delineates the research landscape on social determinants of MH in Germany. The findings point towards a predominant focus on individual-level determinants, with comparatively limited attention to systemic and structural factors. Moreover, an emphasis on mental illness rather than MH, alongside substantial heterogeneity in measured constructs, may constrain the understanding of MH disparities. These gaps highlight the need for more comprehensive, intersectional approaches that account for the diversity of individuals, contexts, and outcomes.

**Supplementary information:**

The online version contains supplementary material available at 10.1186/s12939-026-02877-0.

## Introduction

Mental health (MH) is an important public health issue and perspective due to the high prevalence of mental illnesses, which are associated with frequent comorbidities, substantial individual suffering, and considerable societal costs. Accordingly, awareness of MH has steadily increased over time. The first formal definition, formulated in 1948, described MH as a “condition that enables the optimal physical, intellectual, and emotional development of the individual, as long as this is compatible with the development of others” [1]. In 2004, the WHO expanded the concept to include well-being, emphasizing self-actualization and positive emotional states rather than the mere absence of illness [1]. Notably, the concept of MH has evolved over time and is still subject to change [2, 3]. The most up-to date definition of MH was introduced in 2022 by the WHO as “a state of mental well-being that enables people to cope with the stresses of life, recognize their abilities, learn and work well, and contribute to their communities” [4, 5]. However, critics have repeatedly pointed out the incompatibility of this definition with real-life struggles, as it tends to categorize ordinary crises and adaptive coping mechanisms as signs of mental illness (MI) and risks “blaming thevictim” for their social circumstances [1]. Although there is still much debate surrounding the nosological concept of MH, a study [6] from 2002 suggested, that MH may be more adequately conceptualized as part of a continuum from languishing to flourishing. Since then, many studies have adopted this idea, and subsequently developed it into the dual-continuum model, which posits that MH and MI are distinct but interrelated constructs, each existing along a continuum [7]. While MI is typically attached to psychiatric disorders, common indicators of MH include life satisfaction, positive affect, mental balance, and overall wellbeing.

Observed differences in MH outcomes across population groups may be partly explained by the influence of various social determinants (SD) [8]. Research on social determinants of health (SDoH) builds on the Whitehall Study, led by Michael Marmot, who famously asked: “Why treat people and send them back to the conditions that made them sick in the first place?”. SDoH are defined as “the conditions in which people are born, grow, live, work and age” [9], and are often grouped into five key domains: economic stability, education access and quality, healthcare access and quality, neighborhood and built environment, and social and community context [10, 11]. MH has also been included in the UN Sustainable development goals, underlining its importance as a global public health priority, highlighting proximal factors as immediate environment, and distal factors as structural societal influences [12]. However, considerable heterogeneity in the literature regarding the terminology and taxonomy of SDoH still remains. More recent conceptualizations also incorporate environmental and climate change as domains, arguing that a healthy environment is essential for healthy communities [13]. This perspective is often referred to as planetary health, closely linked with MH [14]. Some studies also refer to non-medical factors that influence health outcomes, including the development, prevention, and severity of various health conditions [15]. Other studies also consider health-related knowledge, beliefs, and behaviors, such as smoking, as factors that may mediate the relationship between SDoHs and health outcomes [16]. The WHO [9] further emphasizes that health inequalities often arise from the unequal distribution of power, money, and resources.

Over the past years, studies have consistently demonstrated that SDoH exert substantial and harmful effects on MH through inequitable social conditions [17]. For example, educational attainment can predict depression, food insecurity is linked to depression, anxiety, and sleep disorders, and difficulties with housing correlate with worse MH [17]. Lund et al. [18] reviewed existing evidence and found that female gender is associated with an increased risk of major depressive disorder, while poverty is consistently associated with an increased prevalence of depression and anxiety disorders, and living in an urban environment is associated with schizophrenia [18]. Bhugra & Becker [19] emphasized that migration experience and discrimination are pronounced risk factors for MI, especially depression and anxiety disorders. In the same realm, the experience of stigma related to MI was found to affect individuals’ perspectives and MH-related behaviors such as help-seeking [20, 21]. Studies often employ separate instruments to measure MH and MI [22]. For MH outcomes, Lund et al. [18] found, for example, that housing insecurity, food insecurity, and financial instability were associated with life dissatisfaction. Another study demonstrated that factors as age, ethnicity, health, education, and housing had an impact on ‘happiness’; household composition and ‘couple display’ affected the estimation of one’s ‘fulfilled life’ [23].

Importantly, SDoH interact with one another rather than functioning in isolation. Intersectionality, introduced by Crenshaw [24] in 1989, helps to explain how multiple social categories combine to create unique experiences of privilege or disadvantage. Although still rare in public health research, applying intersectionality offers valuable insights for improving health outcomes [25]. Consequently, stigma and discrimination experiences related to MI have been integrated into models of MH causation [17, 26, 27]. Accordingly, the WHO [28] calls for strategies that move beyond individual behaviors and address the broader societal and systemic factors shaping MH outcomes.

### The case of Germany

In Germany - a high-income nation characterized by a robust social welfare system - inequalities in social security and living conditions continue to be evident [29]. Germany has long lagged behind other high-income countries in terms of life expectancy, which is often attributed to demographic change, i.e., the ‘ageing population’ [30, 31]. Around 14% of the population lives in poverty [29] and mental disorders are a significant public health concern [32], contributing to high morbidity, straining healthcare resources, and resulting in substantial social and economic costs. For example, according to OECD data, the direct and indirect costs of MH conditions in Germany were estimated at around €146.5 billion in 2015, corresponding to approximately 4.8% of the country’s gross domestic product. Moreover, nearly one in five people in Germany experienced a MH problem in 2019, highlighting the high prevalence and broad societal impact of these conditions [33]. In order to take a closer look at the SDoH of Germany, several large-scale surveys were carried out since the 1980s [34], targeting different populations. For example, the German Health Update (GEDA) collects nationwide data on health status, behaviors, and healthcare use among adults since 2009, while the German Health Interview and Examination Survey for Children and Adolescents (KiGGS), since 2003, focuses on physical health and MH in young people. The German Emigration and Remigration Panel Study (GERPS) examines the causes and consequences of international mobility among German nationals–an important area, given high psychological distress among asylum seekers and refugees in Germany [35]. Finally, the Study of Health in Pomerania (SHIP) investigates health risk factors in Northeast Germany, taking into account the situation of Germany after the reunification [36].

### Objectives

To the best of our knowledge, no study to date has examined the overall landscape of SDoH research in the German context. This presents a valuable opportunity to compile existing findings within this framework and identify key concepts. We aimed to map the landscape of SDoH research, addressing the context of Germany, focusing on three key questions: (1) Which SDoH are most or least frequently researched? (2) What health outcomes are most examined in terms of MH or MI? (3) Which theoretical frameworks and explanatory mechanisms are used and tested to explain the link between SDoH and MH outcomes, and is intersectionality considered? By systematically mapping the existing literature, this scoping review provides an overview of how SDoH and MH outcomes have been studied in the German context. It characterizes the distribution of research across different SDoH, MH outcomes, and conceptual approaches, thereby highlighting which areas have received substantial attention and where empirical evidence remains limited. Through this structured overview of the research landscape, the review contributes to a clearer methodological-focused understanding of the research on MH disparities in Germany and identifies directions for future research.

## Methods

The methodology of the present systematic scoping review followed the guidelines of the Preferred Reporting Items for Systematic Reviews and Meta-Analyses extension for Scoping Reviews (PRISMA-ScR) [37].

### Protocol and registration

The protocol was established by the research team and prospectively registered with the Open Science Framework on December 17, 2023 (10.17605/OSF.IO/DSH89). As no human participants were involved in this study, ethical approval was not required.

### Eligibility criteria

To be included in this scoping review, publications needed to meet the following inclusion criteria: (i) population: individuals of all ages and all genders of the general population living in Germany, given that we were interested in a broad overview of SDoH researched in Germany, (ii) studies that (for this population) deal with the influence of SDs on MH and MI, to gain a more comprehensive understanding of social influences on the full spectrum of mental well-being, (iii) quantitative empirical studies with validated data collection instruments (i.e., randomized controlled trials, cohort studies, case-control studies; measurement instruments: validated self-assessment and external assessment procedures, diagnostic procedures with/without structured interviews, register data), to ensure better comparability between the different studies, and (iv) publication: published in peer-reviewed German or English scientific journals with an abstract of each publication available online to reflect common quality standards and ensure comprehensibility for the research team.

We excluded: (i) studies that look exclusively at populations with pre-existing mental or physical health conditions, (ii) studies that only look at physical illnesses/complaints; studies that do not investigate the relationship between SDoH and MH/MI, (iii) qualitative studies, field studies, experiments; measurement instruments: non-validated instruments, and (iv) publication: published neither in German nor in English; type: books, book chapters, book reviews, commentaries, corrections, editorials, introductions, forewords, letters, statements, dissertations, lectures, posters.

### Information sources

To identify relevant literature, a comprehensive keyword-based electronic database search was conducted in the online databases PubMed and Web of Science.

### Search

Our database literature search was conducted on January 23rd, 2024 (and updated on April 17, 2025). The search strategies were drafted and refined through discussion by the research team. The keyword search included natural language terms and Medical Subject Headings (MeSH), where applicable, related to (1) SDoHs in conjunction with (2) Germany. Titles and abstracts were searched using the terms (soci* determinant* of health) AND Germany[Title/Abstract] in PubMed, and (TS=(soci* determinants of health)) AND (CU=(Germany)) in Web of Science. Given our interest in peer-reviewed studies as a common measure of quality assurance, no further measures were taken to identify gray literature.

### Selection of sources of evidence

The final search results were imported into Covidence (https://www.covidence.org), a web-based systematic review tool, and duplicates were removed by the software and hand. Three reviewers (ML, GH, PR) independently performed the title, abstract, and full text screening against the inclusion and exclusion criteria. Two independent decisions were required per each title/abstract and full text. Disagreements on study selection and data extraction were resolved by consensus upon team discussion. To increase the consistency between reviewers, all reviewers met regularly at the beginning of the screening process, discussed their potential conflicting understanding of the inclusion and exclusion criteria based on ambiguous decisions on a certain literature entry, and amended the screening and data extraction procedure before independently continuing to screen for this review.

### Data charting process

A data-charting form was jointly developed by the group of reviewers (ML, GH, PR), to determine which data to extract to answer the research questions. The form was implemented in Covidence, and the data of each publication had to be charted independently by two reviewers. In case of discrepancy, conflicting data extractions were discussed, and the finalized extracted data was agreed upon in consensus team meetings.

### Data items

We extracted data on publication characteristics (e.g., title, reference, publication year, publication type, funding sources, and conflicts of interest), study characteristics (e.g., type of study, study design, and aim of study), sample and participant information (e.g., sample size, gender, age, ethnicity, and education), recruitment process (e.g., start and end of data collection, inclusion and exclusion criteria, recruitment method), assessed SDoHs, outcome measures, theoretical frameworks and explanatory mechanisms (i.e., assumed variables in the relationship between SDoH and MI/MH as well as theoretical concepts about the relationship), applied statistical methods, key results, and authors´ conclusions regarding SDoH.

### Synthesis of results

To categorize the spectrum of SDoH addressed in the literature, we conducted a content analysis using a combined deductive-inductive approach. We first reviewed existing SDoH frameworks [9, 38–40], which differ substantially in the number, scope, and level of abstraction of SDoH domains. While some conceptualizations vary mainly in the breadth and number of domains, others introduce hierarchical structures (e.g., micro-, meso-, and macro-level determinants). Based on this review, we deductively developed an initial SDoH domain category framework to guide the classification process through critical expert discussion within the research team. Given the aim of mapping the heterogeneous landscape of SDoHs examined in the literature, we adopted a broader concept-based classification rather than implementing a more complex hierarchical structure. Two reviewers then independently assigned the extracted data on assessed SDoH constructs to the predefined SDoH domain category system. During this process, the category system was iteratively refined through inductive adjustments based on the content of the included studies and further expert discussion within the research team. The resulting classification (for details, please refer to the Results/Researched SDoHs section) was subsequently analyzed quantitatively.

Regarding the reported outcomes, we identified the assessed constructs along with their operationalizations with validated measures, grouped them into higher-order categories (i.e., *assessed construct*: specific mental disorders, general behavior/mood, general health/quality of life, health behavior, and other; *assessment focus*: MH vs. MI; *assessment procedure*: self-rated vs. objective/expert-rated), and quantitatively assessed the field, e.g., regarding the number of outcomes assessed per study, the assessed constructs, the assessment’s focus, and the use of self-report versus objective/expert-rated measures.

Regarding the third research question, we distinguished between two aspects: (1) “theoretical frameworks & explanatory mechanisms”, which captures conceptual approaches, and the terminology used in the studies, and (2) “intersectionality considered”. Regarding the consideration of intersectionality, we also considered statistical interactions between two or more variables as intersectionality, since this reflects the overlapping influence of multiple SDs. Although socio-economic status (SES) combines multiple dimensions such as education, occupation, and income, it was treated as an aggregated indicator of socioeconomic position rather than an intersectional construct, as it does not clearly capture interactions between distinct social categories and the forms of assessment vary substantially between included studies. Data were synthesized narratively for this third research question.

Data regarding age, sex/gender, ethnicity, and education were extracted and, if applicable, computed to means or percentage values per study for better comparability.

## Results

### Study selection

**Fig. 1:**
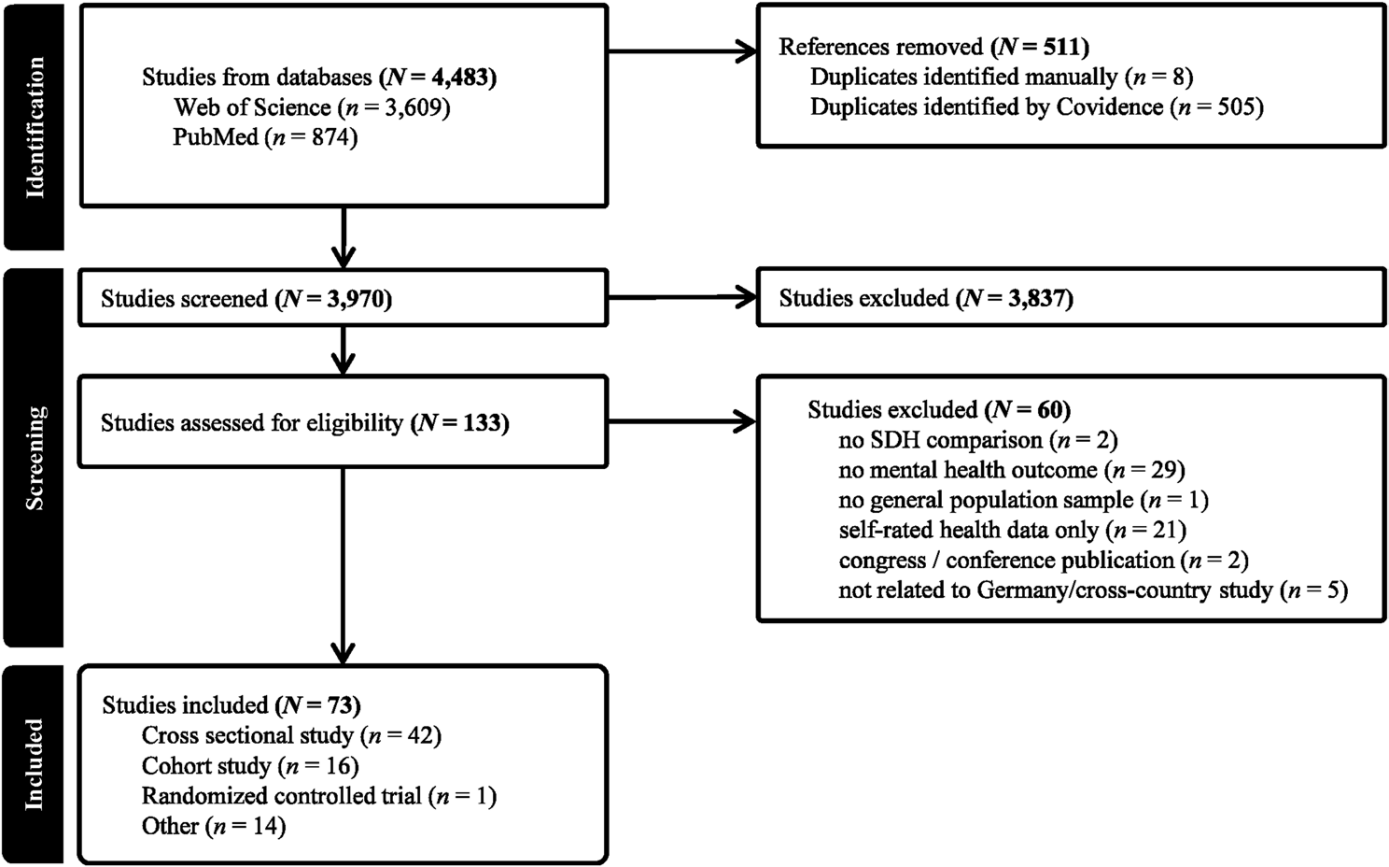
PRISMA Flow chart

A total of 4,483 records were identified from PubMed and Web of Science. After removal of duplicates and records that did not meet the inclusion criteria or met the exclusion criteria, 73 studies were included in the final synthesis. Figure 1 shows the PRISMA Flow Chart and reasons for exclusion at each step of the screening process. The characteristics of the included studies are presented in Table 1.

**Table 1:** Design, sample size and characteristics, assessed social determinants of health, assessed outcomes and operationalizations, underlying theoretical frameworks & explanatory mechanisms, and the consideration of Intersectionality in included studies

Study	Design	Sample Size (N)	Years of Age (Mean)	Gender (% female)	Ethnicity	Education	Assessed Social Determinants of Health^a^	Assessed Outcomes and Operationalizations^b^	Theoretical frameworks and explanatory mechanisms	Intersectionality considered
Adedeji et al., 2023	Cross sectional study	518	32.5	38.9	100.0% sub-Saharan African migrants in Germany	1.2% none, 17.0% secondary/elementary, 31.7% vocational school certificate, 33.2% university degree, 17.0% master/technician/equivalent degree	DEM, ICC, EDU	quality of life (WHOQOL-BREF, psychological domain)	Migration related stress, social capital, acculturation stress	*Intersectionality*: no; *Interaction*: no
Aichberger et al., 2012	Cross sectional study	405	40.7	100.0	N/A; 49.4% native German; 50.6% Turkish	18.4% low education, 30.8% high education	DEM, ICC, ECO, EDU	emotional distress (GHQ- 28)	Migration related stress, income inequality and a continuously increasing gap between social groups in Europe (SES); minority group; social exclusion	*Intersectionality*: no; *Interaction*: education ×employment
Barbek et al., 2024	Cross sectional study	2,413	N/A	51.1	77.4% migration history	N/A	DEM, ICC, ECO, OTH	health anxiety (WI-7)	Intersectionality Theory, health anxiety, social inequalities, privileges	*Intersectionality*: in introduction (Black feminist theory: disadvantage, discrimination, inequality); *Interaction*: yes
Bartig et al., 2023	Cross sectional study	6,038	39.0	49.4 (weighted 46.2)	N/A; nationality: 26.2% Turkish, 21.5% Polish, 18.9% Italian, 18.0% Croatian, 15.4% Syrian	45.6% low education, 40.1% medium education, 14.3% high education	DEM, ICC, EDU	depressive symptoms (PHQ-9)	History of migration, circumstances of migration, health risks, discrimination, psychological stressors, subjective health	*Intersectionality*: only mentioned in conclusion *Interaction*: no
Bartig et al., 2023	Cross sectional study	6,038	39.0	49.4 (weighted 46.2)	N/A; nationality: 26.2% Turkish, 21.5% Polish, 18.9% Italian, 18.0% Croatian, 15.4% Syrian	45.6% low education, 40.1% medium education, 14.3% high education	DEM, ICC, ECO, EDU	depression diagnosis (self-constructed yes/no/NA-single-item measure)	Migration history, health resources and disease risks, discrimination, social exclusion	*Intersectionality*: no; *Interaction*: no
Bau et al., 2011	Cross sectional study	1,842	12.3	100.0	N/A; 30.6% migration background; 9.1% one-sided, 24.8% both-sided migration background	younger girls (10 - 12 years): 66.7% primary school, 33.3% grammar school. Older girls (13 - 15 years): 49% grammar school, 23% secondary school, 20% comprehensive school, 2% primary school, 6% secondary school.	DEM, ICC, OTH	HRQoL (KINDL-R)	HRQol of life of children and adolescents; risk and protective factors; social status	*Intersectionality*: no; *Interaction*: no
Becker-Gruenig et al., 2016	Cross sectional study	1,106	9.1	48.7	N/A; 18.0% migration background	N/A	DEM, ICC, ECO, NBE	HRQoL (KINDL-R), behavioral problems (SDQ)	Social-ecological model (Bronfenbrenner), socio-demographic factors, macro- and micro-context factors, individual lifestyle and biographical factors	*Intersectionality*: no; *Interaction*: no
Belau et al., 2021	Cross sectional study	208	36.5	32.7	41.8% Syrian, 10.6% Afghan, 27.9% Iraqi, 3.8% Iranian, 6.7% African, 9.1% Other	N/A	DEM, ICC	mental health problems (SF-12-SOEP, MCS score)	Psychological stress and coping theory (Lazarus), conservation of resources theory (Hobfoll), refugees-related loneliness and loss, family separation	*Intersectionality*: no; *Interaction*: no
Beutel et al., 2002	Cross sectional study	2,182	48.5	0.0	N/A	less than 10th grade: 49.7% (sample 1), 48.8% (sample 2); completed 10th grade: 27.6% (sample 1), 31.0% (sample 2); completed high school:11.7% (sample 1), 11.3% (sample 2); college or university degree: 11.0% (sample 1), 9.9% (sample 2)	DEM, ICC, ECO, EDU, NBE	fatigue (MFI-20), anxiety and depressive symptoms (HADS-D)	Biological and psychosocial determinants, “midlife crisis”, psychosocial vulnerability and protective factors, fatigue, “ageing male”	*Intersectionality*: no; *Interaction*: no
Beutel et al., 2004	Cross sectional study	2,771	48.6	100.0	N/A	52% less than 10th grade, 36% 10th grade, 5% high school, 7% college/university	DEM, ICC, ECO, EDU, NBE	fatigue (MFI-20), anxiety and depressive symptoms (HADS-D)	Age-related complaints in women, psychosocial vulnerability and protective factors, ‘‘empty nest’’, “aging woman”	*Intersectionality*: no; *Interaction*: no
Beutel et al., 2017	Cohort study	15,010	54.9	49.4	N/A	N/A	DEM, ICC, ECO, NBE	depressive symptoms (PHQ-8), anxiety (GAD-2), suicidal ideation (PHQ-9)	Loneliness, social integration, health behavior, a “need to belong”	*Intersectionality*: no; *Interaction*: no
Biddle et al., 2024	Other: natural experiment design using longitudinal data from three waves of the IAB-SOEP-BAMF Refugee Panel	1,466	N/A	36.8	43.5% Syria, 17.6% Iraq, 15.4% Afghanistan, 9.1% other Asia, 10.6% Africa, 3.8% other	64.6% low, 18.1% medium, 17.3% high	DEM, ICC, ECO, EDU, NBE	change (t1-t0) in mental and physical health scores (SF-12v2)	Socio-economic deprivation, contextual factors, marginalized groups	*Intersectionality*: no; *Interaction*: no; Mediation
Blume et al., 2024	Other: German Health Update: Fokus (GEDA Fokus)”	6,038	N/A	49.4	N/A; Croatian, Italian, Polish, Syrian, Turkish	N/A	DEM, ICC, ECO, ENV	depressive symptoms (PHQ-9), symptoms of Generalized Anxiety Disorder (GAD-7)	Individual and structural risk, mental stress, intersectionality theory	*Intersectionality*: only mentioned in introduction, *Interaction*: no
Bolster et al., 2025	Cohort study	4,707	34.0	84.4 mothers	17.6% not born in Germany	Education:0.5% primary education, 13.6% lower secondary, 85.3% upper secondary	DEM, ICC, ECO, EDU, OTH	depressive symptoms (PHQ-2), anxiety symptoms (GAD-2), double burden of having depressive and anxiety symptoms (PHQ-4)	Neighborhood effects, vulnerable group, family mental health	*Intersectionality*: “interaction effects of stress, migration background, financial worries, and education level on the association between neighborhood SES and parental mental heal; *Interaction*: stress, migration background, financial worries, education level as moderators
Borgmann et al., 2019	Cross sectional study	2,062 single mothers; 242 single fathers	N/A	87.1	N/A	N/A (only reported as combined index)	DEM, ICC, EDU, NBE	HRQoL (CDC HRQOL-4, number of emotionally distressed days), 12-month prevalence of diagnosed depression (self-reported)	Parenting, chronic stress, social support, double burdens, structural conditions	*Intersectionality*: no; *Interaction*: no
Buchcik et al., 2021	Cross sectional study	755	39.6 (Migrants), 51.6 (Native Germans)	58.5 (Migrants), 55.0 (Native Germans)	37.6% non-German/migrants	Migrants: 1.1% none, 3.2% 1–5 years, 23.6% 6–10 years, 28.5% 11–13 years, 25.4% >13 years, 18.3% not specified; Natives: 0.0% none, 1.3% 1–5 years, 38.4% 6–10 years, 26.5% 11–13 years, 27.0% >13 years, 6.8% not specified	DEM, ICC, ECO, EDU	HRQoL (SF-12)	Migration background, focus on minority groups, discrimination, health inequality, social support, integration	*Intersectionality*: no; *Interaction*: no
Chae et al., 2022	Cross sectional study	7,987 (DEGS1-MH: 4493)	N/A	51.1	N/A	N/A (included in SES variable)	DEM, ICC, ECO, EDU	depression diagnosis (CIDI-MDD), depressive symptoms (PHQ-9)	Public health, risk factors, co-occurrence of Depression and obesity, stigma	*Intersectionality*: no; *Interaction*: obesity ×sex and age group ×sex and in the conclusion
Cohrdes et al., 2018	Cross sectional study	3,667	52.7	52.0	N/A	N/A (included in SES variable)	DEM, ICC, ECO, OTH	mental health problems (SF-36, MCS score)	Supportive factors of HRQoL (Self-reported HRQoL), individual, social and lifestyle factors	*Intersectionality* no; *Interactions*: age group × physical activity as well as age group × healthy eating interactions
Eibich et al., 2016	Cohort study	2,200	60.8	53.0	N/A	on average 14.4 years of education	DEM, ICC, NBE, ENV, HAQ	physical and mental health (SF-12)	Neighborhood environment, local policy, social capital	*Intersectionality*: no; *Interaction*: age group ×social support
Erhart et al., 2024	Other: Socio-Economic Panel (SOEP)	19,994	48.4	48.6	N/A, 32.5% migration background	17.9% low education, 47.7% medium education	DEM, ICC, EDU	depressive symptoms (PHQ-4)	Intersectionality Theory, Power Theories, Structural Theory, suggesting that socioeconomic status influences health across the lifespan, Resilience theory	*Intersectionality*: A intersectional Multilevel Analysis was employed *Interaction*: between the social categories
Georges et al., 2023	Cohort study	25,659	N/A	52.1	N/A; 5.1% migration background	9.0% ISCED1+2; 52.2% ISCED3; 6.3% ISCED4; 8.4% ISCED5; 24.2% ISCED6	DEM, ICC, EDU	mental health (SF-12, MCS score)	Vulnerability, pre-, peri- and post-migration status, post-caregiving health models (Corey Magan), risk-resilience models and stress models	*Intersectionality* was defined as: Migration status, caregiving; *Interaction*: migrant status ×caregiving
Giesebrecht et al., 2024	Cross sectional study	144	31.9	33.3	47.9% Iran, 16.0% Afghanistan, 12.5% Syria, 6.9% Somalia, 5.6% Eritrea, 2.1% Algeria, 9.1% other countries/missing information	12.5% no completion of formal schooling, 8.3% primary school, 14.6% secondary school, 51.4% school-leaving examination or higher, 13.2% missing information	DEM, ICC, EDU	symptoms of posttraumatic stress (PDS), depressive symptoms (PHQ-9)	Chronic stress due to discrimination, social identity, protective factors, moderating factor of social support, ethnic and institutional discrimination, marginalized group	*Intersectionality*: only mentioned in the discussion:” *Interaction*: yes
Grochtdreis et al., 2024	Other: cross-sectional and longitudinal study using secondary data analysis of data from German Socio-Economic Panel (SOEP)	4,124	38.8	50.6	Ethnicity (country of birth): 11.7% Russia, 8.9% Romania, 8.8% Kazakhstan, 11.7% Turkey, 9.8% other East European country, 21.8% other European country, 3.7% African country, 21.2% other Asian country, 2.4% American/Oceanic country	3.66% no school-leaving qualification, 35.85% secondary general school, 26.37% secondary school, 29.57% academic secondary school	DEM, ICC, ECO, EDU, ENV	health-related quality of life (SF-12v2)	HRQoL, discrimination, disadvantage, post migration stressors	*Intersectionality*: no; *Interaction*: no
Hajek et al., 2016	Cohort study	2,443	82.6	66.0	N/A	60.9% low, 27.6% middle, 11.5% high	DEM, ICC, EDU	HRQoL (EQ-5D visual analogue scale mean)	HRQoL, vulnerability hypothesis, social support	*Intersectionality* no; *Interaction*: social support ×gender
Hajek et al., 2021	Cross sectional study	952	85.8	45.0	N/A	21.6 low, 53.0% medium, 25.3 high	DEM, ICC, ECO, EDU	depressive symptoms (DIA-S)	Loneliness, social network, successful aging among the oldest old	*Intersectionality*: no; *Interaction*: no
Hajek et al., 2022	Cohort study	648	88.8	67.8	N/A	56.4% primary, 29.4% secondary, 14.2% tertiary	DEM, ICC, EDU	HRQoL (EQ-5D-3 L)	HRQoL; social support, social network, oldest old	*Intersectionality*: no; *Interaction*: no
Hajek et al., 2023	Cohort study	1,760	86.6	49.6	N/A	20.8% low, 53.7% medium, 25.5 high	DEM, ICC	depressive symptoms (DIA-S4)	Oldest old, loneliness	*Intersectionality*: no; *Interaction*: no
Hajek et al., 2023	Cross sectional study	104 transgender people	30.4	N/A	11.1% migration background	52.5% absence, and 39.4% presence of general or subject-specific university entrance qualification; 8.1% missing data	DEM, ICC, ECO, EDU, OTH	depressive symptoms (PHQ-9), generalized anxiety disorder symptoms (GAD-7)	Gender affirmation, risk factors	*Intersectionality*: no; *Interaction*: no
Hammersen et al., 2016	Cross sectional study	19,294	N/A	51.7	N/A	N/A	DEM, ICC, ECO, NBE, ENV	mental health (MHI-5, subscale of SF-36)	Transtheoretical stress mode (Lazarus), environmental noise, biological, psychological, Social, economic, and environmental factors,	*Intersectionality*: no; *Interaction*: no
Hetzel et al., 2016	Cross sectional study	3,176	67.3 (men), 66.1 (women)	40.2	N/A	27.6% none, 36.0% vocational school, 25.9% completed apprenticeship, 10.5% college/university	DEM, ICC, ECO	mental health impairments	Disengagement theory, activity theory, demand-resource model, salutogenesis, self-efficacy, subjective well-being	*Intersectionality*: no; *Interaction*: no
Hoebel et al., 2013	Cross sectional study	2,827	N/A	51.4	N/A	35.6% low, 33.3% medium, 29.8% high	DEM, ICC, ECO, EDU	HRQoL (4 items from ALLBUS survey)	Socially disadvantaged groups, vulnerability	*Intersectionality*: no; *Interaction*: no
Hoebel et al., 2017	Cross sectional study	4,952	49.9	55.9	N/A	3.5 score on a scale from 1 to 7	DEM, ICC, ECO, EDU	depressive symptoms (PHQ-8)	Social comparison processes, fair or unfair opportunities in life, social inequality, social stratification (Weber), subjective social status	*Intersectionality*: no; *Interaction*: no; Mediation
Hollederer et al., 2016	Other: panel study	8,845	42.4	49.8	13.5% non-German	Education:36.9% low, 42.5% medium, 20.5% high	DEM, ICC, ECO, EDU, NBE	subjective health status, mental health problems, number of contact to medical professionals and hospitals, physical exercise (Items from PASS panel study)	“Poverty and unemployment make people ill”, theory of action restriction (Fryer), psychological deprivation among the unemployed (Jahoda), stress theories for coping with the life phase of unemployment, identity theories	*Intersectionality*: no; *Interaction*: no
Horsfield et al., 2020	Other: Cross sectional and longitudinal studies	12,785	N/A	N/A	N/A	N/A (see John et al. (2001) and Völzke et al. (2011))	DEM, ICC, ECO, EDU	unspecified somatic and mental health complaints (BL-38)	Physical and psychological complaints, risk factors	*Intersectionality*: no; *Interaction*: no
Karl et al., 2024	Other: Cohort study/panel	3,455	31.4	57.9	3.6% born in country other than Germany	26.2% below or 10 years, 73.8% more than 10 years	DEM, ECO	somatization, anxiety, obsessive-compulsiveness, hostility (SCL-90-R); postpartum depression (EPDS)	Precarious employment	*Intersectionality*: no; *Interaction*: no
Kirkcaldy et al., 2004	Cross sectional study	980	15.6	51.8	N/A	N/A	DEM	suicidal ideation, self-injurious behavior/intentions	Risk Factors, social and psychological predictors	*Intersectionality*: no; *Interaction*: gender × age
Kroll et al., 2016	Cross sectional study	18,465	N/A	0.0	N/A	N/A	DEM, ICC, ECO, EDU, NBE	single items from panel survey asking for mental health problems (number of days within last 30 days), health awareness (general care for own health, caring for sufficient physical exercise), health behavior (physical exercise, smoking)	Work-life balance, risk behavior, causation thesis, selection thesis, multiple role attachment hypothesis	*Intersectionality*: no; *Interaction*: employment ×parental, and partner status
Kroll et al., 2016	Cross sectional study	31,955	N/A	N/A	N/A	N/A	ECO	depression diagnosis by medical professional	Prevention projects for the unemployed, risk of unemployment, protective health behavior, causation thesis, selection thesis, and composition effects	*Intersectionality*: no; *Interaction*: no
Kuehne et al., 2015	Cohort study	96	37.0	72.0	58.3% Latin American or Caribbean, 17.7% African, 16.7% from Southeast- or East-Europe or countries formerly belonging to the Soviet Union, 7.3% Others	N/A	DEM, ICC	HRQoL (SF-12v2)	Vulnerable population, discrimination, subjective health, migration history, HRQoL, barriers	*Intersectionality*: no; *Interaction*: no
Laemmle et al., 2013	Cross sectional study	4,529	9.4	49.5	N/A	N/A	ICC, ECO, EDU, NBE	psychosomatic complaints (single rating items on headache, stomachache, abdominal pain, backache, and chest pain) and subjective health (single rating item)	Biopsychosocial model (Engel), risk factors, theory of triadic influence (Flay & Petraitis), Integrated Change Model, health behavior	*Intersectionality*: no; *Interaction*: of a multitude of socio-economic; Mediation
Limm et al., 2012	Cross sectional study	365	43.7	57.8	N/A; 36.2% migration background	33.3% <10 years, 32.2% 10–11 years, 34.4% ≥12 years	DEM, ICC, ECO, EDU, ENV, OTH	mental health (SF-12, MCS score)	Risk factors, bi-directional relationship between unemployment and health, appraisal theory, subjective health	*Intersectionality*: no; *Interaction*: no
Lohaus et al., 2017	Cross sectional study	219	4.1	52.5	N/A	N/A	DEM, ICC	mental health problems (CBCL)	Parental stress, bidirectional relationship between parental stress and child behavior problems, social support,	*Intersectionality*: no; *Interaction*: no
Lotty et al., 2015	Cross sectional study	2,352	N/A	54.8	35.1% EU-member states (without Germany), 16.5% African, 12.5% European states (neither EU nor GUS member), 6.9% Asian, 5.5% former GUS-states	N/A	DEM, ICC, NBE	ICD diagnosis, extent of treatment	Gaps in care, barriers	*Intersectionality*: no; *Interaction*: no
Lüschen et al., 1997	Cross sectional study	2,554	47.2	53.5	0.0% non-German	*M* = 10.9 years of education	DEM, ICC, ECO, EDU	health status (subjective health status, psychological distress, multimorbidity as the sum of 11 different diseases, sickness in the last year, cardio-vascular diseases, skeleton and muscle impairments), physician utilization; specific item information/wording not specified in manuscript	System integration, social stratification	*Intersectionality*: no; *Interaction*: no
Massag et al., 2023	Cohort study	13,934	N/A	60.1	N/A; 2.7% not born in Germany, 0.2% missing data	3.5% low, 29.2% medium, 63.1% high	DEM, ECO, ENV	depressive symptoms (PHQ-9), anxiety symptoms (GAD-7), distress (modified PDI)	Mass traumatic events (war), consequences, trauma, habituation	*Intersectionality*: no; *Interaction*: no
Meyrose et al., 2018	Other: Longitudinal data from panel studies	2,810	12.2	48.7	N/A; 9.5% migration background	maternal 22.2% low, 62.1% medium, 15.7% high	DEM, ICC, ECO, EDU, OTH	mental health (parent-reported SDQ)	Risk-groups, mental health trajectories, bioecological approach (Bronfenbrenner), social inequality, “the childhood-limited model”	*Intersectionality*: no; gender-, age-, and maternal education-specific trajectories;moderator maternal education (time × gender, time × age, time × gender × age, time × maternal education, gender × age, gender × maternal education, age × maternal education, family structure × maternal education)
Morales et al., 2022	Other: methodological study	20,828	N/A	N/A	N/A	N/A	DEM, ECO, NBE, HAQ	prevalence of major depression	Regional prevalence	*Intersectionality*: no; *Interaction*: no
Mugambwa et al., 2023	Cross sectional study	1,071	N/A	42.8	80.0% non-German; 39.9% EU/EEA countries without Germany; 40% non-EU countries	N/A	DEM, ICC, ECO, NBE, HAQ	perceived altered mental health (participants’ self-report on perceived depression, lack of interest, and moral support), diagnosed mental disorders (clinician-coded ICD-10 diagnoses)	Limited access to regular health services, barriers, vulnerable groups, Trauma related stress, migration trajectories, social exclusion and marginalization, stigma	*Intersectionality*: no; *Interaction*: no
Naeher et al., 2020	Cohort study	149,033	N/A	N/A	N/A	N/A	DEM, ICC, ECO, EDU, OTH	suicide coded according to ICD-9 and ICD-10	Suicide ideation, psychological model of suicide (Joiner)	*Intersectionality*: no; *Interaction*: SES × SI
Petrowski et al., 2021	Cross sectional study	3,020	49.0	54.0	0.0% non-German	N/A	DEM, ECO, ENV	depressive symptoms (PHQ-2), general anxiety symptoms (GAD-2), self-esteem (RSES), general life satisfaction (FLZM)	Risk and protective factors, biopsychosocial impacts of air pollution, chronic stress, allostatic load theory, adaptive cost hypothesis, resources to cope with environmental demands may interfere with other adaptive processes (e.g., psychosocial stressors), subjective well-being	*Intersectionality*: no; *Interaction*: no
Pfoertner et al., 2022	Other: The study uses cross-sectional and longitudinal data from the German Socio-Economic Panel (GSOEP)	38,551	N/A	49.4	8.4% non-German	27.1% low, 45.2% moderate, 27.7% high	DEM, ICC, ECO, NBE	mental and physical health (SF-12v2)	Dual Labor Market Theory, insider–outsider theory, unstable and insecure nature of precarious employment in the labor market is typically a persistent state and form of a social class of “the precariat”, life course, mobility trajectories in precariousness of employment and physical and mental health, stress-related illness	*Intersectionality*: no; *Interaction*: precariousness of employment ×gender; mobility in precariousness of employment ×gender
Pinker et al., 2021	Cross sectional study	1,428	34.5	100.0	N/A; 9.6% migration background	0.1% non, 7.4% low, 32.1% medium, 60.1% high, 0.4% other	DEM, ICC, ECO, EDU, NBE, HAQ, OTH	HRQoL (EQ-5D visual analogue scale; SF-12 PCS and MCS subscores)	Risk factors; protective factors, health is multidimensional, social support	*Intersectionality*: no; *Interaction*: of a multitude of socio-economic variables
Poethko-Müller et al., 2024	Cross sectional study	9,766	59.0	53.4	N/A	14.9% low, 43.4% medium, 41.5% high	DEM, ICC, EDU, OTH	fatigue (FAS)	Prevalence, inequality	*Intersectionality*: no; *Interaction*: no
Rattay et al., 2020	Cross sectional study	39,096	N/A	49.1	0.0% non-German	22.4% low, 61.6% middle, 16.0% high	DEM, ICC, ECO, NBE	self-rated general health (question “How is your general state of health?”), depression (whether respondent was suffering from depression or depressive mood in the last 12 months diagnosed by a doctor or psychotherapist), back pain (12-month prevalence; whether the respondent has had at least three months of persistent back pain in the last 12 months), overweight (based on respondents’ height and weight data), smoking (question “Do you smoke from time to time—even if only occasionally?”), sporting inactivity (respondents’ self-declaration that they have not practiced any sport in the last 3 months)	Social role of parenthood, emerging adulthood, “causality” hypothesis, the health and health behavior of women and men can also have an influence on the probability of finding a partner or starting a family (“selectivity” hypothesis), gender comparative studies, welfare states	*Intersectionality*: no; *Interaction*: parental status ×age
Ravens-Sieberer et al., 2009	Other: methodological/modeling study on three existing datasets	16,700	N/A	N/A	N/A	N/A	ICC, ECO	HRQoL (KINDL-R), emotional and behavioral problems (SDQ), functional limitations and need for additional services due to chronic health conditions (CSHCN)	HRQoL: a multidimensional construct that encompasses physical, emotional, mental, social, spiritual, and behavioral components of well-being and functional capacity (ability to act) from a subjective perspective, subjective health status, well-being; social resources, subjective assessment, salutogenic approaches, resilience	*Intersectionality*: no; *Interaction*: no
Ravens-Sieberer et al., 2012	Cohort study	11,619	N/A	50.3	N/A	N/A	DEM, ECO, EDU	HRQoL (KIDSCREEN-10), mental health difficulties (SDQ)	Public health measures, health promotion and prevention measures, HRQoL, Children and adolescents’ living environment (e.g., family, school), school stress	*Intersectionality*: no; *Interaction*: no
Reuter et al., 2023	Cross sectional study	3,214	20.9	45.0	4.7% non-German	60.1% low education, 39.9% high education	DEM, ICC, ECO, EDU, NBE	self-rated health (“How would you describe your general state of health?”), number of days with a health event in the past year, list of seven symptoms of musculoskeletal disorders (i.e., pain in back, neck/shoulder, arms, hand, hips, knees, legs/feet; constructed a sum score) and five symptoms of mental health problems (i.e., sleep disturbances, tiredness/faintness/fatigue, nervousness/irritability, low mood, emotional exhaustion; constructed a sum score)	Health inequalities, socio-economically stratified, Job demands, job insecurity	*Intersectionality*: no; *Interaction*: no; Mediation
Rothermund et al., 2003	Other: cross-sectional and 8-year longitudinal research format	1,256	N/A	50.6	N/A	N/A	DEM, ICC, ECO, OTH	depressive symptoms (GDS)	Resilience, external factors (increasing strain or stress) to internal factors (loss of coping resources, vulnerabilities), or both. TGP measures a disposition to tenaciously maintain personal goals even against obstacles (assimilative tenacity), whereas FGA assesses the readiness to flexibly adjust goals and ambitions to constraints and impairments (accommodative flexibility), control and learned helplessness formulations (Seligman)	*Intersectionality*: no; *Interaction*: interactions of gender ×cohort or measurement occasion
Santos-Hövener et al., 2019	Cross sectional study	13,568	N/A	50.2	N/A; 9.2% one-sided, 10.3% both-sided migration background; 1.2% missing data	12.3% low, 60.9% medium, 25.2% high	DEM, ICC, ECO	mental health (SDQ), ADHD symptoms (SDQ subscale), BMI, allergic illnesses	Migration background, health inequalities, barriers in the healthcare system	*Intersectionality*: no; *Interaction*: no
Schaffrath et al., 2024	Cross sectional study	189	31.5	30.2	100% migration background; 29.1% Syria, 15.3% Afghanistan, 7.9% Iran, 7.4% Iraq, 6.3% Albania, 4.2% Serbia, 3.2% Macedonia, 2.6% Kosovo, etc.	*M* = 9.53 years of education and vocational training	DEM, ICC, ECO, ENV	patient status (yes/no); PTSD symptoms (HTQ), depressive symptoms (PHQ-9), global burden (SCL-K9), utilization of psychiatric-psychotherapeutic treatment	Post-migration social stress factors, trauma, chronicity	*Intersectionality*: no; *Interaction*: no
Scharte et al., 2013	Cross sectional study	17,218	N/A	53.5	10.0% non-German	N/A	DEM, ICC, ECO, EDU, ENV	mental health (SDQ)	Differences are explained by socio-economic factors. Question of causality and reverse effects, vulnerable group	*Intersectionality*: no; *Interaction*: yes
Schilz et al., 2023	Randomised controlled trial	325	30.6	30.8	100.0% non-German; Arabic, Farsi	*M* = 8.53 years of schooling	DEM, ICC, EDU, ENV	depressive symptoms (PHQ-9), PTSD symptoms (HTQ), HRQoL (WHOQOL BREF)	Vulnerable groups, multiple stressors, capability approach as a framework to understand the impact of displacement-related stressors on forcibly displaced people, protective factors	*Intersectionality*: no; *Interaction*: no
Schunck et al., 2015	Other: longitudinal panel survey	2,851	46.6	51.0	61.0% non-German	1% in school, 33% inadequately completed/general elementary, 36% middle vocational, 9% university entrance degree, 6% higher vocational, 16% higher education	DEM, ICC, ECO, EDU	physical and mental health (SF-12)	Discrimination as stressor, differential vulnerability, reverse causality	*Intersectionality*: no; *Interaction*: countries of origin ×perceived discrimination
Schwager et al., 2020	Other: longitudinal study in the context of a pilot study	163	13.4	41.5	N/A	43.3% fourth to sixth grade, 31.1% seventh to eighth grade, 25.6% tenth to eleventh grade	DEM, ICC	mental and physical well-being (KINDL-R)	Sociometer theory, need to belong (Baumeister& Leary), adolescence as a vulnerable group	*Intersectionality* no; *Interaction*: no; Mediation
Schwarz et al., 2007	Cross sectional study	1,119	45.5	100.0	N/A	N/A	DEM, ICC, ECO, EDU	HRQoL/general health symptoms (ZSL), depression prevalence	Lifestyle, women’s health	*Intersectionality*: no; *Interaction*: no
Soeder et al., 2025	Cross sectional study	1,105	25.5	50.1	N/A, 18.8% migration background	67.2% university student, 32.8% university of applied sciences student	DEM, ICC, ECO, EDU, NBE, ENV	sleep difficulties (JSS), well-being (WHO-5), depressive symptoms (PHQ-8)	Climate change as threat, climate change worry, stress	*Intersectionality*: no; *Interaction*: no
The BELLA Study Group et al., 2008	Cross sectional study	2,863 families; 1,903 children and adolescents	N/A	N/A	N/A	N/A (unit of analysis is families)	DEM, ICC, ECO, OTH	behavioral problems (SDQ)	“Millennial morbidity” (dated to the period between 2000 and the present), salutogenetic approach (Aaron Antonovsky), protective factors, personal resources, resilience, social support	*Intersectionality*: no; *Interaction*: interaction of risk and protective factors
The BELLA Study Group et al., 2015	Cohort study	1,643	13.9	50.6	N/A	N/A	DEM, ICC, ECO, EDU	depressive symptoms (CES-DC)	Resilience, risk factors and protective factors, resources, family system, social support	*Intersectionality*: no; *Interaction*: interaction of risk and protective factors
Vonneilich et al., 2011	Cohort study	4,814	59.6	50.2	N/A	11.4% ≤10 years; 55.6% 11–13 years; 22.2% 14–17 years	DEM, ICC, ECO, EDU	depressive symptoms (CES-DC)	Different vulnerability, socially deprived, socioeconomic health inequalities across different societies, differential exposure hypothesis, differential vulnerability hypothesis	*Intersectionality*: no; *Interaction*: no
Waldhauer et al., 2018	Cohort study	4,665	N/A	49.6	N/A	38% secondary/high school, 58% elementary school	DEM, ICC, ECO, EDU	mental health and behavioral problems (SDQ)	Class atmosphere, personal resources, individual and structural learning conditions, educational opportunities, educational capital, inequalities	*Intersectionality*: no; *Interaction*: no
Walther et al., 2020	Cohort study	2,639	N/A	36.6	N/A; nationality: 53.4% Syrian, 12.6% Afghan, 12.1% Iraqi, 6.5% Eritrean, 15.4% other	59.6% low, 21.0% medium, 19.4% high	DEM, ICC, EDU	psychological distress encompassing symptoms of depression, anxiety and PTSD (RHS-13)	Risk factors, psychological distress, context-specific, vulnerable groups, postmigration challenges	*Intersectionality*: no; *Interaction*: no
Wuestner et al., 2019	Cohort study	1,384	13.9	51.0	N/A; 4.0% migration background	N/A	DEM, ICC, ECO	ADHD symptoms (CGI-P), depressive symptoms (CES-DC), generalized anxiety symptoms (SCARED-D)	Risk and protective factors in the context of ADHD, family climate	*Intersectionality*: no; *Interaction*: risk and protective factors
Zeier et al., 2024	Cross sectional study	871	49.3	56.4	N/A	89.1% college or higher	DEM, ENV	psychological distress regarding general anxiety and depression (PHQ-4)	Environment, according to theories on affect, emotions are key antecedents of human behavior, eco-emotions, adaptive and maladaptive aspects of these emotions	*Intersectionality*: no; *Interaction*: no

Note. In the intersectionality column, we also considered statistical interactions between two or more variables as intersectionality, since this reflects the overlapping influence of multiple social determinants

HRQoL = Health-related quality of life; PTSD = Post-traumatic stress disorder; ADHD = Attention deficit hyperactivity disorder; BMI = Body mass index; ALLBUS = Allgemeine Bevölkerungsumfrage der Sozialwissenschaften (English: German General Social Survey, GGSS); PASS = Panel Arbeitsmarkt und soziale Sicherung (English: Panel Labor Market and Social Security)

^a^Social Determinants of Health-related abbreviations include: DEM = Demographic; ICC = Interpersonal, community, and cultural; ECO = Economic/Economic stability; EDU = Education; NBE = Neighborhood and built environment; ENV = Environmental events; HAQ = Healthcare access and quality; OTH = Other health-related determinants

^b^Measurement instrument-related abbreviations include: BL-38 = BL-38 subjective complaints scale; CBCL = Child Behavior Checklist; CDC HRQOL-4 = Centers for Disease Control and Prevention health-related quality of life core set; CES-DC = Center for Epidemiologic Studies Depression Scale for Children; CGI-P = Conners’ Global Index–Parent Version; CIDI-MDD = Composite International Diagnostic Interview–Depression Module; CSHCN = Children with Special Health Care Needs Screener; DIA-S = Depression In Old Age Scale (Depression im Alter-Skala); DIA-S4 = Depression In Old Age Scale (Depression im Alter-Skala) – Short form 4; EPDS = Edinburgh Postnatal Depressions Scale; EQ-5D = EuroQol Quality of Life 5 Dimensions; EQ-5D-3 L = EuroQol Quality of Life 5 Dimensions 3 Level Version; FAS = Fatigue Assessment Scale; FLZM = German version of the General Life Satisfaction Questionnaire (FLZM—Allgemeine Lebenszufriedenheit); GAD-2 = Generalized Anxiety Disorder 2-item; GAD-7 = Generalized Anxiety Disorder Scale-7; GDS = Geriatric Depression Scale; GHQ-28 = General Health Questionnaire–28; HADS-D = German version of the Hospital Anxiety and Depression Scale; HTQ = Harvard Trauma Questionnaire; JSS = Jenkins Sleep Scale; KIDSCREEN-10 = Short form of KIDSCREEN-27 for the assessment of HRQoL in children and adolescents; KINDL-R = Questionnaire for Measuring Health-Related Quality of Life in Children and Adolescents (Revidierter Fragebogen für KINDer und Jugendliche zur Erfassung der gesundheitsbezogenen Lebensqualität); MFI-20 = Multidimensional Fatigue Inventory; MHI-5 = Mental Health Inventory-5; PDI = Peritraumatic Distress Inventory; PDS = Posttraumatic Diagnostic Scale; PHQ-2 = Patient Health Questionnaire-2; PHQ-4 = Patient Health Questionnaire-4; PHQ-8 = Patient Health Questionnaire-8; PHQ-9 = Patient Health Questionnaire-9; RHS-13 = Refugee Health Screener-13; RSES = Rosenberg Self-Esteem Scale; SCARED-D = Screen for Child Anxiety-Related Emotional Disorders (German version); SCL-90-R = Symptom Checklist-90-Revised; SCL-K9 = Symptom Checklist Short Form-9; SDQ = Strengths-and-Difficulties Questionnaire; SF-12 = Short Form 12; SF-12-SOEP = Short Form-12 Health Survey-SOEP; SF-12v2 = Short Form-12 version 2; SF-36 = Short Form-36; WHO-5 = WHO-5 Well-being Index; WHOQOL-BREF = World Health Organization Quality of Life–BREF (Short form); WI-7 = Whiteley Index-7; ZSL = Von Zerssen Symptom List

### Researched social determinants of health

Our first research question focused on identifying which SDoH are most and least frequently studied. To address this, we analyzed how often each SDoH domain was represented across the 73 included studies - that is, how many studies assessed at least one construct of the respective domain. The most frequently examined domains were ‘Demographics’ (DEM; *n* = 72, 96.0%), ‘Interpersonal, Community, and Cultural’ (ICC; *n* = 67, 89.3%), ‘Economic/Economic Stability’ (ECO; *n* = 54, 72.0%), and ‘Education’ (EDU; *n* = 43, 57.3%). In contrast, domains such as ‘Neighborhood and Built Environment’ (NBE; *n* = 19, 25.3%), ‘Environmental Events’ (ENV; *n* = 12, 16.0%), ‘Healthcare Access and Quality’ (HAQ; *n* = 4, 5.3%), and ‘Other Health-related Determinants’ (OTH; *n* = 12, 16.0%) were addressed less frequently.

A more detailed analysis based on the absolute numbers of assessed constructs per SDoH domain revealed a slightly different picture. The domain ‘Interpersonal, Community, and Cultural’ (*n* = 179, 32.4%) ranked first, followed by ‘Demographics’ (*n* = 151, 27.4%). The domain ‘Economic/Economic stability’ (*n* = 85, 15.4%) remained in third place, while ‘Education’ (*n* = 44, 8.0%) ranked fourth. The remaining domains were less frequently represented in the included studies: ‘Other Health-related Determinants’ (*n* = 43, 7.8%), ‘Neighborhood and Built Environment’ (*n* = 26, 4.7%), ‘Environmental Events’ (*n* = 19, 3.4%), and ‘Healthcare Access and Quality’ (*n* = 5, 0.9%).

Next, we examined the distribution of constructs within each SDoH domain. Figures 2, 3, 4, 5, 6, 7, 8, 9 show, in aggregate, the relative frequency (in %) of constructs within the respective category. Detailed data by study is available in Supplementary Table S1.

**Fig. 2:**
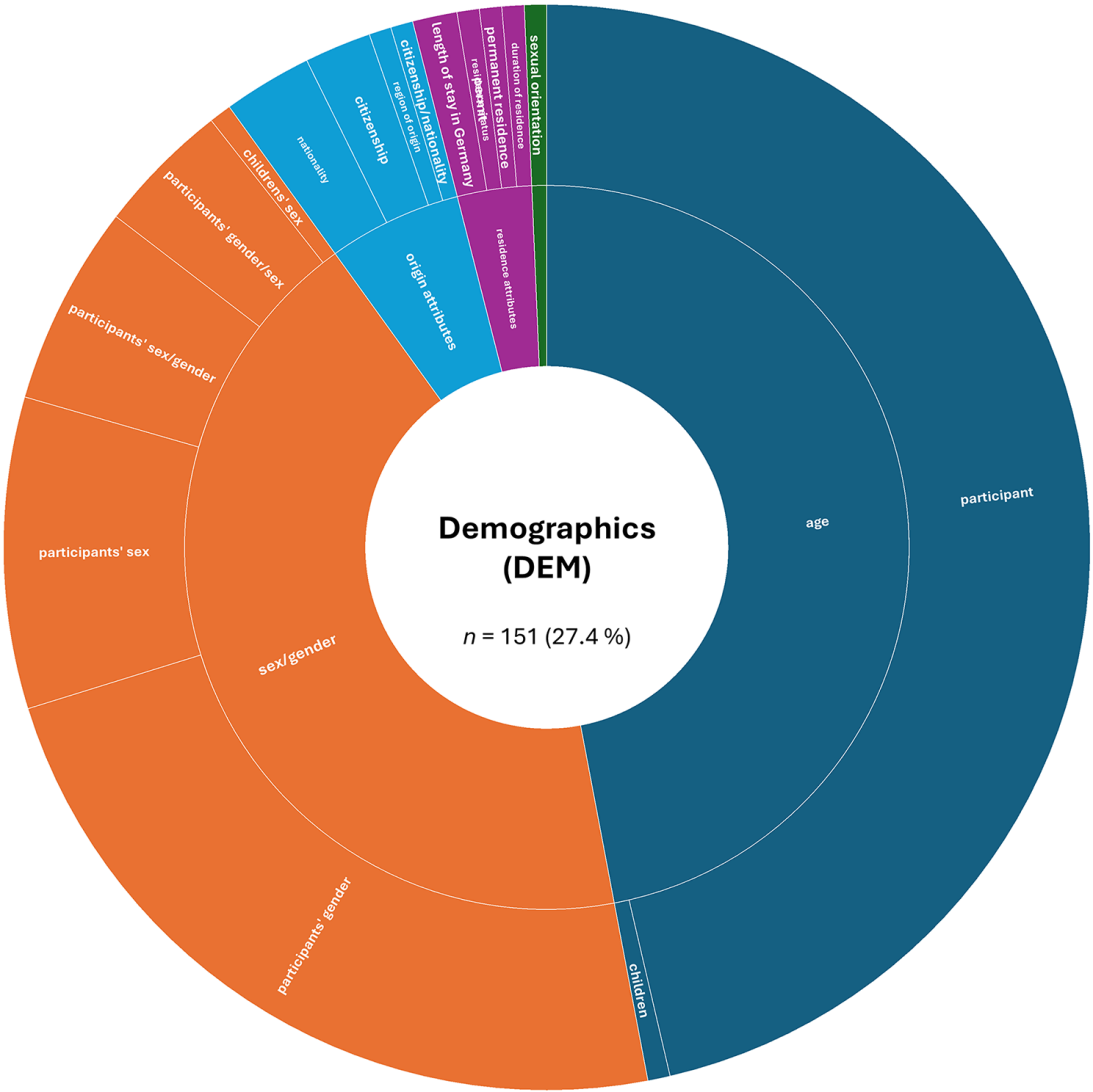
Assessed social determinants of health categorized as demographics

**Fig. 3:**
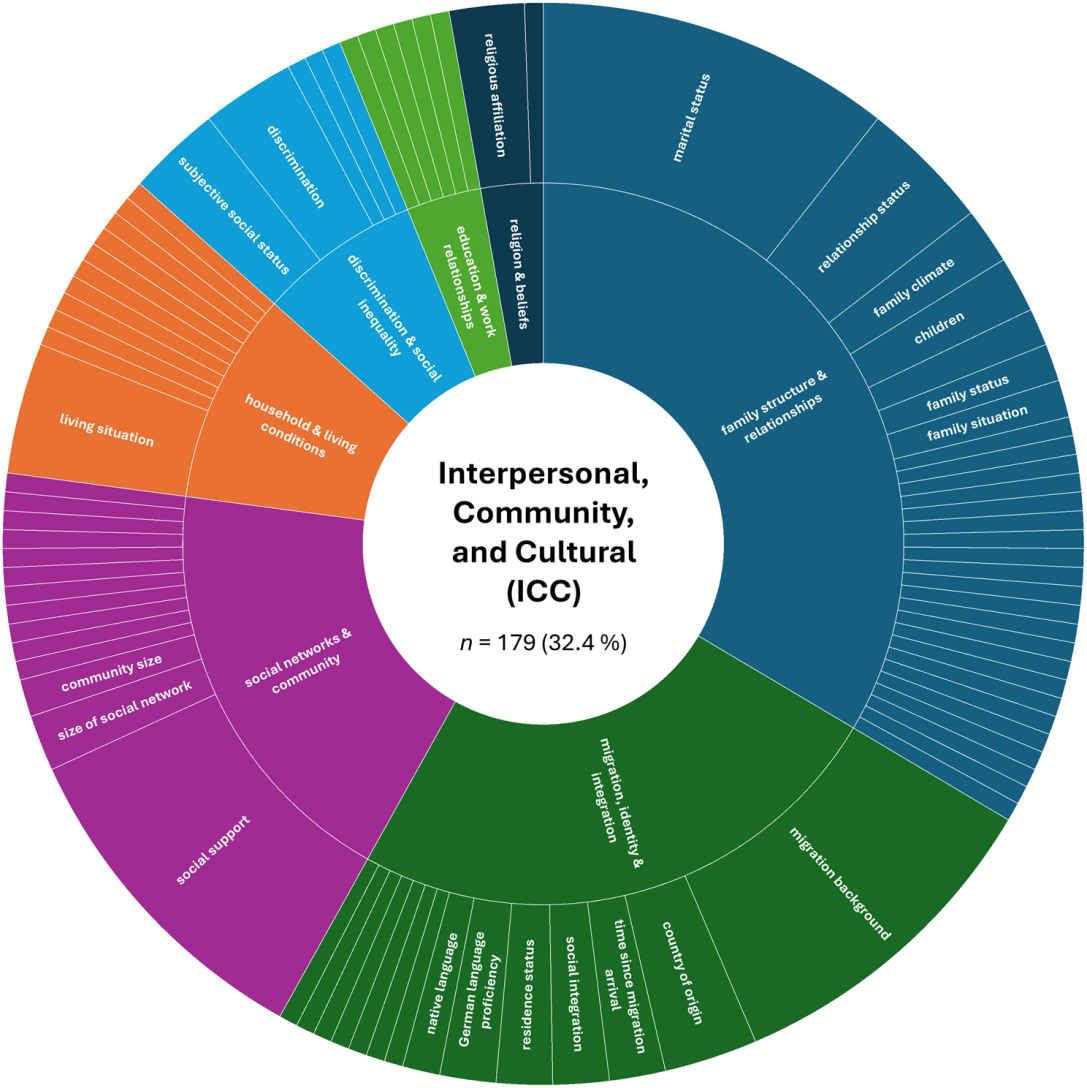
Assessed social determinants of health categorized as Interpersonal, community, and cultural

**Fig. 4:**
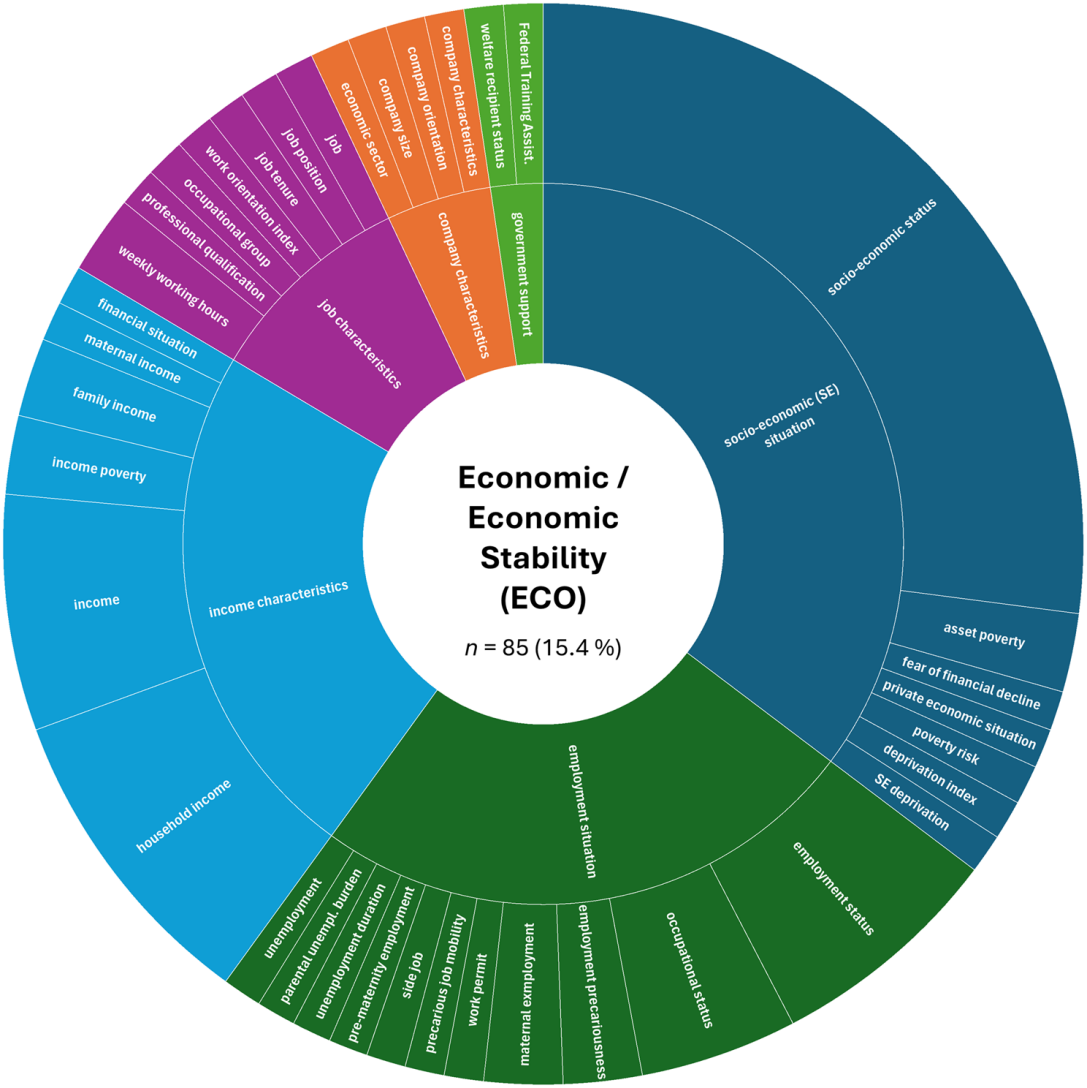
Assessed social determinants of health categorized as economic or economic stability

**Fig. 5:**
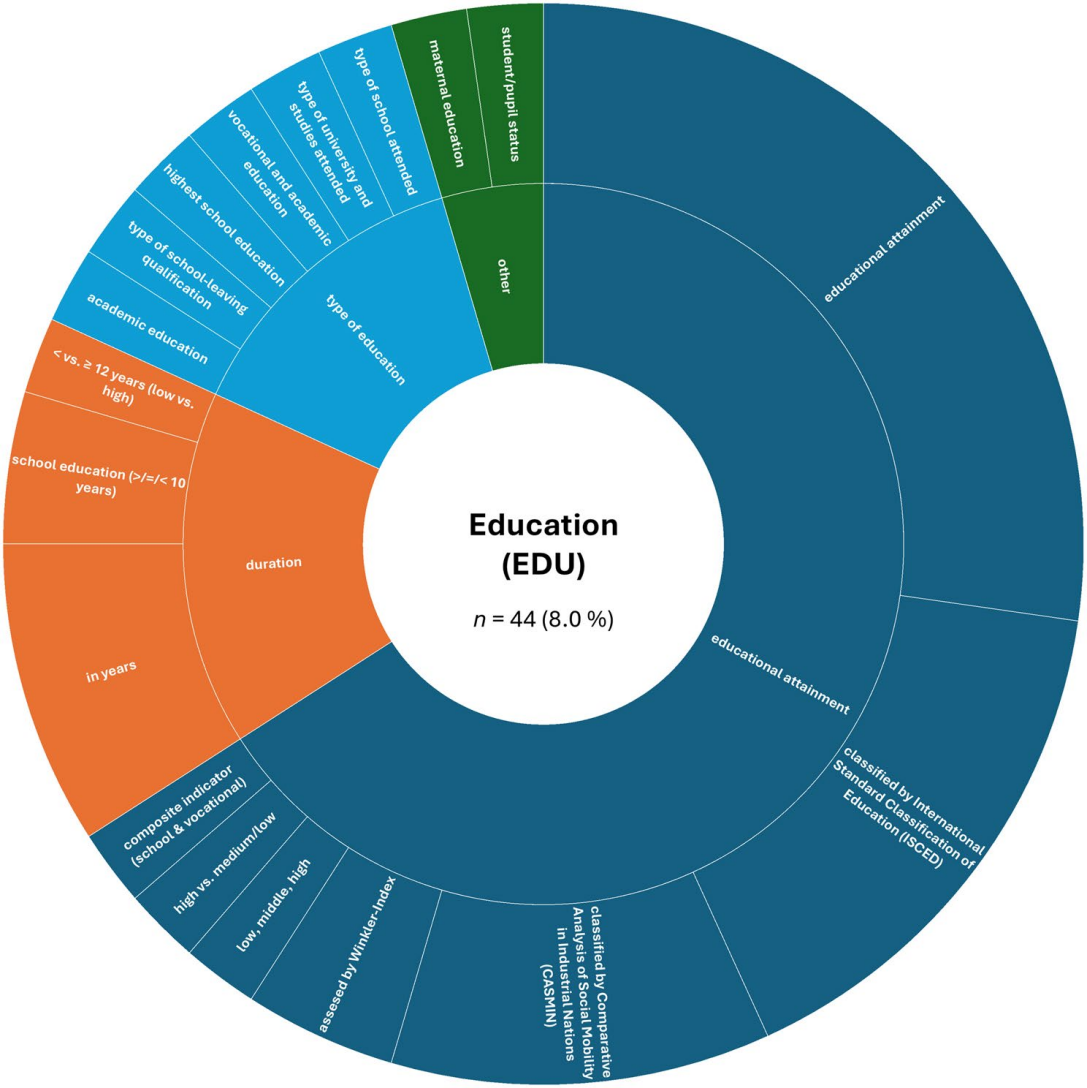
Assessed social determinants of health categorized as education

**Fig. 6:**
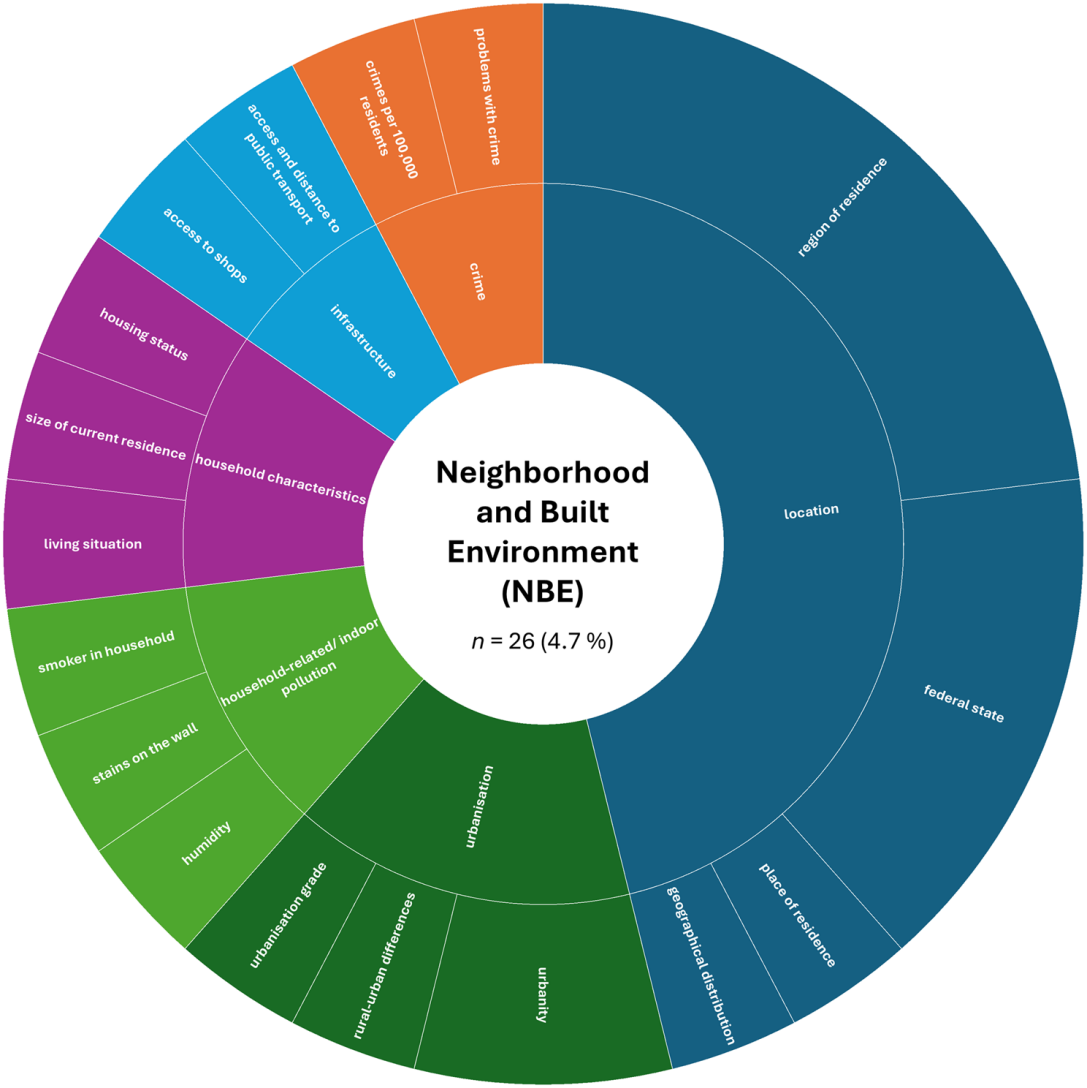
Assessed social determinants of health categorized as Neighborhood and built environment

**Fig. 7:**
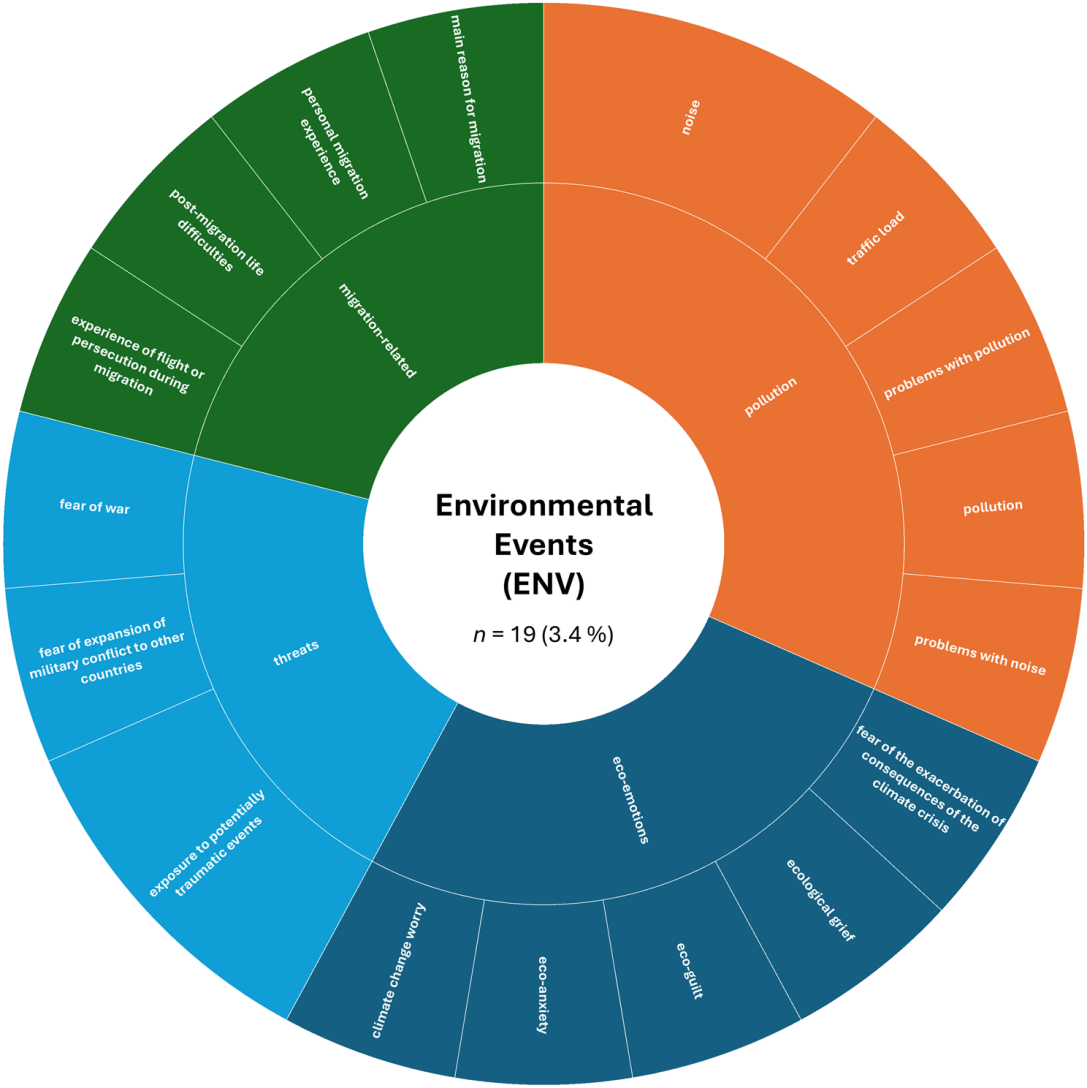
Assessed social determinants of health categorized as environmental events

**Fig. 8:**
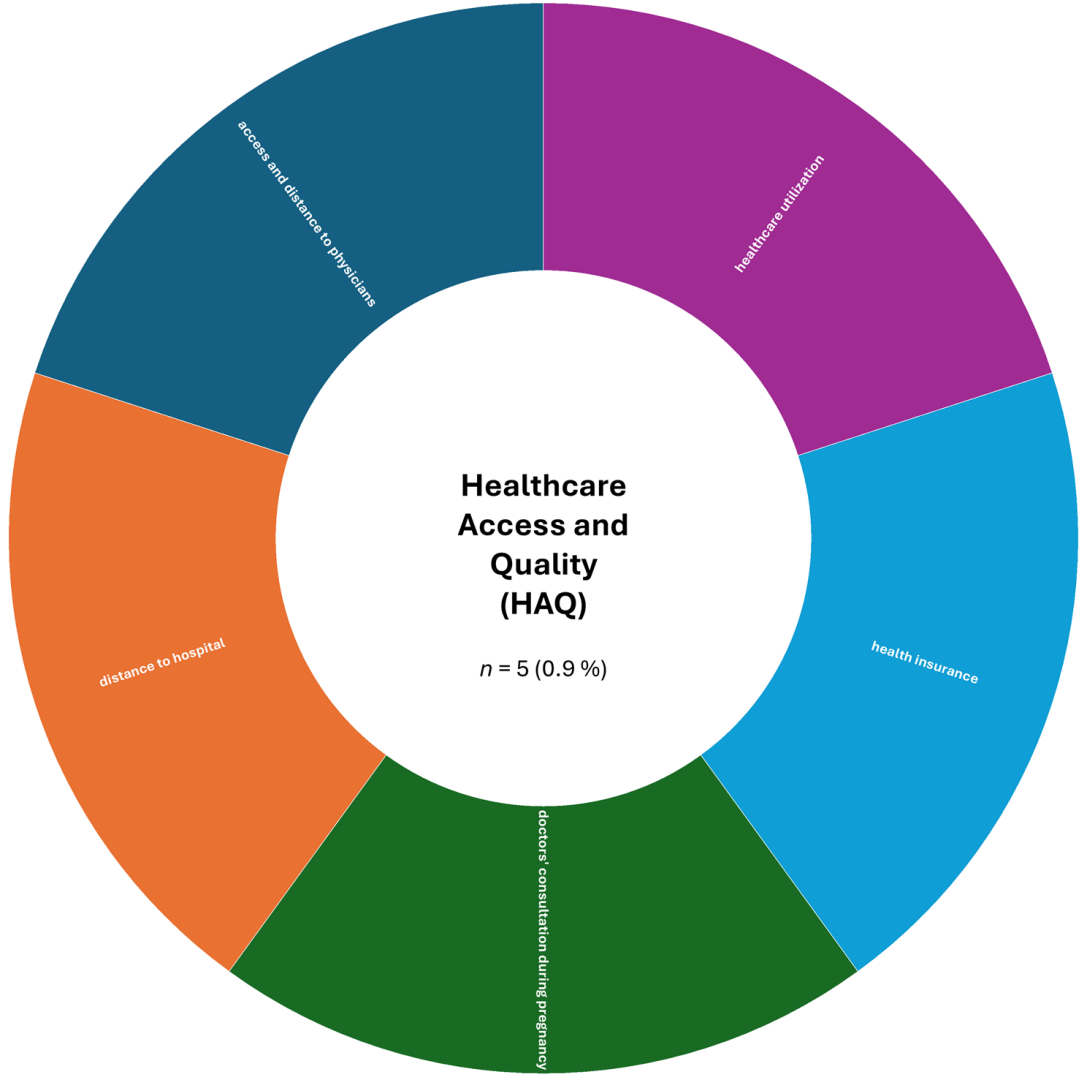
Assessed social determinants of health categorized as healthcare access and quality

**Fig. 9:**
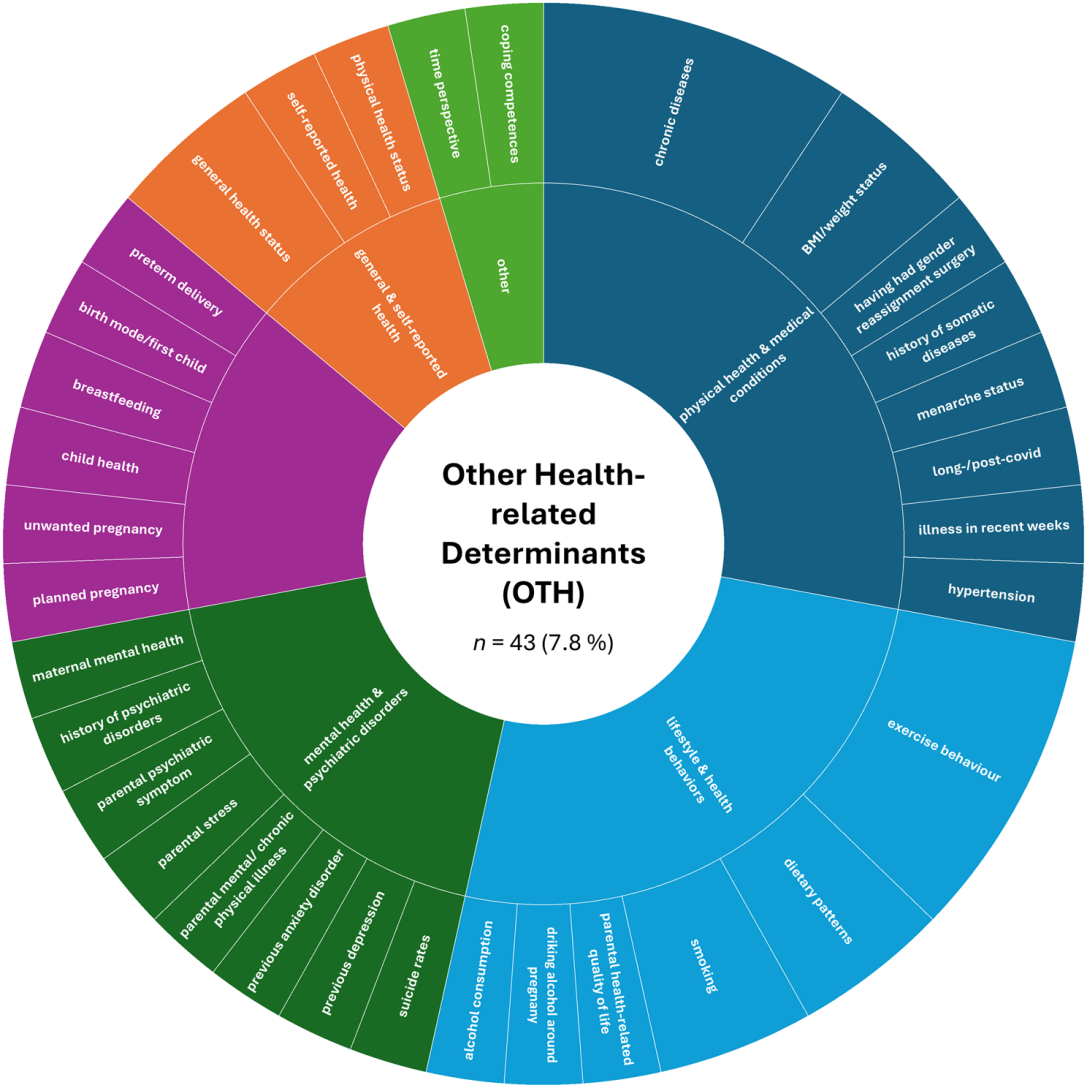
Assessed social determinants of health categorized as other health-related determinants

In the ‘Demographics’ (DEM) domain, the most frequently assessed constructs were age (47.0%) and sex/gender (43.0%). While almost all studies asked for the age of participants (98.6%), one study was particularly interested in age as a specific characteristic of participants’ social environment, asking for the age of their children (1.4%). However, age was often included as a descriptive or control variable, while age-specific analyses (e.g., subgroup analyses or interaction models: *N =* 8), were less frequently conducted. Most studies did not clearly distinguish between sex and gender, often using the terms interchangeably (denoted as “sex/gender” or “gender/sex”; in our tables and figures, we closely follow the terminology employed by the original authors for clarity). A few studies specifically referred to self-reported sex assigned at birth (assigned male/female at birth) or focused on a single gender subgroup (e.g., women, transgender individuals). Notably, most studies employed a binary classification of gender, which may introduce bias by failing to capture the full spectrum of gender identities. The sexual orientation of participants was only assessed in one study (0.7%). Other assessed demographical SDoHs can be grouped into higher-order categories such as ‘origin attributes’ (6.0%; e.g., including citizenship and nationality) and ‘residence attributes’ (3.3%; e.g., duration of residence, residence status, and permit). The least frequently assessed constructs comprised duration of residence, having a permanent residence permit, and nationality (for details, please refer to Fig. 2).

The ‘Interpersonal, Community, and Cultural’ (ICC) domain exhibited substantial heterogeneity. While a few constructs were assessed more often, many were reported only once (for details, please refer to Fig. 3 and Supplementary Table S1), reflecting substantial variability in operationalization within this domain. In terms of higher-order categories, we grouped assessed SDoHs of this domain into ‘family structure and relationships’ (33.5% of all assessments), ‘migration, identity, and integration’ (24.6%), ‘social networks and community’ (19.0%), ‘household and living conditions’ (9.5%), ‘discrimination and social inequality’ (7.3%), ‘education and work relationships’ (3.4%), and ‘religion and beliefs’ (2.8%). Within these higher-order categories, most often assessed constructs included marital status, relationship status, migration background, social support, living situation, subjective social status, and experienced discrimination.

In the ‘Economic/Economic Stability’ (ECO) domain, we again grouped concepts into higher-order categories. These included ‘socio-economic situation’ (35.3% of all assessments), ‘employment situation’ (24.7%), ‘income characteristics’ (23.5%), ‘job characteristics’ (9.4%), ‘company characteristics’ (4.7%), and receiving ‘government support’ (2.4%). The SES was recorded most frequently (27.1% of all assessments within this domain), however its operationalization varied across studies (e.g., based on occupation, education, income). Study-reported variables like socio-economic deprivation, unemployment, maternal income, or the reception of federal training assistance and welfare support were among the less frequently recorded data. Although this domain exhibited less diversity compared to the ‘Interpersonal, Community, and Cultural’ domain, a considerable range of SDoHs was still assessed, the majority of which were reported in only a single study (see Fig. 4 for details).

Regarding the ‘Education’ (EDU) domain, there was relatively little variation regarding the assessment of education across studies. Most studies focused on educational attainment (65.9% of all assessments within this domain), often assessed using standardized classifications such as the International Standard Classification of Education (ISCED) or the Comparative Analysis of Social Mobility in Industrial Nations (CASMIN). Other studies applied simpler categorical groupings (e.g., “low,” “medium,” “high”) or used years of education as a continuous measure. In a considerable proportion of studies, education was assessed as part of a superordinate index such as the Winkler-Index. Education was less frequently assessed in terms of ‘duration’ (15.9%), ‘type of education’ (13.6%), or ‘other’ constructs (4.5%) such as maternal education or student status (see Fig. 5 for details).

In the ‘Neighborhood and Built Environment’ (NBE) domain, most constructs were assessed in only one study, indicating high variability. We again constructed higher-order categories to summarize the domain’s data, including ‘location’ (46.2% of all assessments within this domain; e.g., region of residence), ‘urbanization’ (15.4%; e.g., urbanization grade), ‘household characteristics’ (11.5%; e.g., housing status and size) and ‘household-related/indoor pollution’ (11.5%; e.g., smoking, humidity, stains), as well as ‘infrastructure’ (7.7%; e.g., shops, public transport) and ‘crime’ (7.7%; e.g., crime per 100.000 residents). The most frequently reported assessed constructs were region of residence (Eastern vs. Western), federal state, and urbanity. While region of residence may also be considered a demographic or contextual variable, it was categorized here as a proxy for broader environmental and structural differences, given the enduring structural disparities between Eastern and Western Germany following reunification. All other constructs, including access to transport, crime rates, and housing status, appeared only once (for details, see Fig. 6).

In the ‘Environmental Events’ (ENV) domain, almost all constructs were reported only once, reflecting high heterogeneity. Only noise and exposure to potentially traumatic events were assessed twice. Higher-order categories were identified as ‘pollution’ (31.6% of all assessments within this domain), ‘eco-emotions’ (26.3%), environmental ‘threats’, as well as broader contextual environmental stressors like ‘migration-related’ characteristics (both 21.1%). Assessed constructs ranged from climate-related concerns (e.g., climate change worry, eco-anxiety) to migration-related experiences (e.g., post-migration life difficulties, persecution), and exposure to threats (e.g., fear of war) (refer to Fig. 7 for details).

In the ‘Healthcare Access and Quality’ (HAQ) domain, each construct—such as access to physicians, distance to hospital, health insurance, and healthcare utilization—was assessed in only one study, reflecting both low frequency and limited scope of assessments within this domain (see Fig. 8 for details).

The ‘Other Health-Related Determinants’ (OTH) domain was defined broadly to encompass all additional variables assessed in the included studies that were related to MH or MI. While some of these variables may not constitute SDoHs in a strict sense, they were included due to their classification as health-related factors within the original studies. Consequently, this domain showed substantial heterogeneity, with most constructs assessed in only one study. Among the few more often reported constructs were chronic diseases (9.3% of all assessments within this domain), exercise behavior (9.3%), BMI/weight status, general health status, smoking, and dietary patterns (each of the constructs assessed in 4.7% of the assessments within this domain). Constructed higher-order categories included ‘physical health and medical conditions’ (27.9%), ‘lifestyle and health behaviors’ (25.6%), ‘mental health and psychiatric disorders’ (18.6%), ‘reproductive and child health’ (14.0%), ‘general and self-reported health’ (9.3%), and ‘other’ health-related determinants (4.7%). For details, please refer to Fig. 9.

### Examined outcomes

Regarding the outcomes investigated, the studies present a heterogeneous picture. As shown in Table 2, 50.7% of the included studies assessed mental disorders, while 53.3% focused on aspects of general health or quality of life as outcomes. With a clear gap, general behaviors and moods followed at 18.7%, health behavior at 6.7%, and 10.7% of the studies assessed additional concepts such as psychosomatic and somatic factors, need for assistance, allergies, and other outcomes. Most studies (56.0%) examined only one outcome. Two outcomes were assessed in 24.0% of the studies, three outcomes in 12.0%, four outcomes in 5.3%, and five or six outcomes each in 1.3% of the studies. On average, the studies assessed *M* = 1.67 outcomes (*SD* = 1.09). The majority (92.0%) included at least one self-rated outcome; approximately 24.0% assessed at least one objective or expert-rated outcome, and 16.0% employed a combination of self-rated and expert-rated/objective outcomes.

**Table 2:** Outcomes, researched concepts and utilized measurement instruments

Category	Concept	n^a^	%	Measurement Instruments
Mental Disorders/Conditions		**38**	**50.7**	
	Depression	32	42.7	CES-DC, CIDI-MDD, DIA-S, DIA-S4, diagnosis, EPDS, GDS, HADS-D, PHQ-2, PHQ-4, PHQ-8, PHQ-9, RHS-13, 12-month prevalence
	Anxiety	12	16.0	GAD-2, GAD-7, HADS-D, PHQ-4, RHS-13, SCARED-D, SCL-90-R
	ADHD	2	2.7	CGI-P, SDQ
	Suicide	3	4.0	diagnosis
	Trauma/PTSD	5	6.7	HTQ, PDS, RHS-13
	Fatigue	3	4.0	FAS, MFI-20
	OCD	1	1.3	SCL-90-R
	Health anxiety	1	1.3	WI-7
	Present ICD diagnosis	1	1.3	diagnosis
General Behavior/Mood		**14**	**18.7**	
	Global burden of disease	1	1.3	SCL-K9
	Behavioral problems	7	9.3	SDQ
	Self-esteem	1	1.3	RSES
	Emotional distress	2	2.7	GHQ-28, PDI, number of emotionally distressed days
	Loneliness	1	1.3	single-item measure
	Sleep difficulties	1	1.3	JSS, single-item measure
	Hostility	1	1.3	SCL-90-R
General Health/Quality of Life		**40**	**53.3**	
	General/unspecified mental/physical health	19	25.3	BL-38, CBCL, KINDL-R, MHI-5, SDQ, SF-12 (MCS and PCS subscores), SF-12v2, SF-36, SF12-SOEP (MCS score), self-constructed symptom items, single-item measure
	Life satisfaction	2	2.7	FLZM, single-item measure
	Health-related quality of life	17	22.7	4 ALLBUS items, CDC HRQOL-4, EQ-5D, EQ-5D-3 L, KIDSCREEN-10, KINDL-R, SF-12, SF-12v2, WHO-5, WHOQOL-BREF, ZSL
	Subjective health status	5	6.7	combination of single items
	Health awareness	1	1.3	items from panel study
Health Behavior		**5**	**6.7**	
	Physician utilization	3	4.0	yes/no item, number of contacts
	Patient status	1	1.3	yes/no item
	Smoking	2	2.7	yes/no item
	Sport (in-) activity	3	4.0	single-item measure
Other		**8**	**10.7**	
	Somatic diseases/multimorbidity	2	2.7	yes/no items, single-item measures, diagnosis
	Need for assistance due to functional limitations	1	1.3	CSHCN
	Extent of treatment	1	1.3	self-constructed item
	Body mass index	2	2.7	weight and height data
	Allergic illnesses	1	1.3	single-item measure
	Psychosomatic complaints	1	1.3	SCL-90-R, symptom items, yes/no items
	Back pain	1	1.3	diagnosis, single-item measure
	Number of days with health event in the past year	1	1.3	number of days
Assessment Focus				
	Aspects of Mental Illness	55	73.3	e.g., CES-DC, CGI-P, CIDI-MDD, DIA-S, DIA-S4, EPDS, FAS, GAD-2, etc.
	Aspects of Mental Health	39	52.0	e.g., CDC HRQOL-4, EQ-5D, EQ-5D-3 L, FLZM, WHOQOL-BREF, etc.
	Aspects of both	19	25.3	e.g., SDQ, self-constructed item/assessments, etc.

Note. HRQoL = Health-related quality of life; PTSD = Post-traumatic stress disorder; ADHD = Attention deficit hyperactivity disorder; BMI = Body Mass Index; ALLBUS = Allgemeine Bevölkerungsumfrage der Sozialwissenschaften (English: German General Social Survey, GGSS); PASS = Panel Arbeitsmarkt und soziale Sicherung (English: Panel Labor Market and Social Security)

BL-38 = BL-38 subjective complaints scale; CBCL = Child Behavior Checklist; CDC HRQOL-4 = Centers for Disease Control and Prevention health-related quality of life core set; CES-DC = Center for Epidemiologic Studies Depression Scale for Children; CGI-P = Conners’ Global Index–Parent Version; CIDI-MDD = Composite International Diagnostic Interview–Depression Module; CSHCN = Children with Special Health Care Needs Screener; DIA-S = Depression In Old Age Scale (Depression im Alter-Skala); DIA-S4 = Depression In Old Age Scale (Depression im Alter-Skala) – Short form 4; EPDS = Edinburgh Postnatal Depressions Scale; EQ-5D = EuroQol Quality of Life 5 Dimensions; EQ-5D-3 L = EuroQol Quality of Life 5 Dimensions 3 Level Version; FAS = Fatigue Assessment Scale; FLZM = German version of the General Life Satisfaction Questionnaire (FLZM—Allgemeine Lebenszufriedenheit); GAD-2 = Generalized Anxiety Disorder 2-item; GAD-7 = Generalized Anxiety Disorder Scale-7; GDS = Geriatric Depression Scale; GHQ-28 = General Health Questionnaire–28; HADS-D = German version of the Hospital Anxiety and Depression Scale; HTQ = Harvard Trauma Questionnaire; JSS = Jenkins Sleep Scale; KIDSCREEN-10 = Short form of KIDSCREEN-27 for the assessment of HRQoL in children and adolescents; KINDL-R = Questionnaire for Measuring Health-Related Quality of Life in Children and Adolescents (Revidierter Fragebogen für KINDer und Jugendliche zur Erfassung der gesundheitsbezogenen Lebensqualität); MFI-20 = Multidimensional Fatigue Inventory; MHI-5 = Mental Health Inventory-5; PDI = Peritraumatic Distress Inventory; PDS = Posttraumatic Diagnostic Scale; PHQ-2 = Patient Health Questionnaire-2; PHQ-4 = Patient Health Questionnaire-4; PHQ-8 = Patient Health Questionnaire-8; PHQ-9 = Patient Health Questionnaire-9; RHS-13 = Refugee Health Screener-13; RSES = Rosenberg Self-Esteem Scale; SCARED-D = Screen for Child Anxiety-Related Emotional Disorders (German version); SCL-90-R = Symptom Checklist-90-Revised; SCL-K9 = Symptom Checklist Short Form-9; SDQ = Strengths-and-Difficulties Questionnaire; SF-12 = Short Form 12; SF-12-SOEP = Short Form-12 Health Survey-SOEP; SF-12v2 = Short Form-12 version 2; SF-36 = Short Form-36; WHO-5 = WHO-5 Well-being Index; WHOQOL-BREF = World Health Organization Quality of Life–BREF (Short form); WI-7 = Whiteley Index-7; ZSL = Von Zerssen Symptom List.

^a^Number of studies assessing the respective category or concept.

Along the continuum from MH to MI, 73.3% of the studies examined aspects of MI, while 52.0% considered the perspective of MH. In 25.3% of the studies, outcome measures that captured both perspectives were employed, either through a single instrument or a combination of instruments. Regarding MH, constructs such as general life satisfaction, quality of life, or personal strengths were assessed. In contrast, the MI perspective was often operationalized via psychiatric disorders, days on sick-leave, functional impairments, or personal difficulties. For detailed data on outcomes per each study, please refer to the Table 1.

It should be noted that the studies do not consistently distinguish between these concepts, and the same instrument is sometimes used in one study for the MH and in another for the MI perspective. Regarding the above presented proportions, we revisited the original instrument description to attribute the instrument to either perspective. Health was partly conceptualized as the absence of illness, and partly as the capacity for social participation. Most studies employed psychometrically validated instruments (*N =* 57), while self-developed single-item or combined-item measures were less common. When somatic aspects were included, these were typically assessed separately from psychological aspects, rather than within an integrated psychosomatic understanding.

### Relationship and research concentrations across social determinants and mental health/illness outcomes

**Fig. 10:**
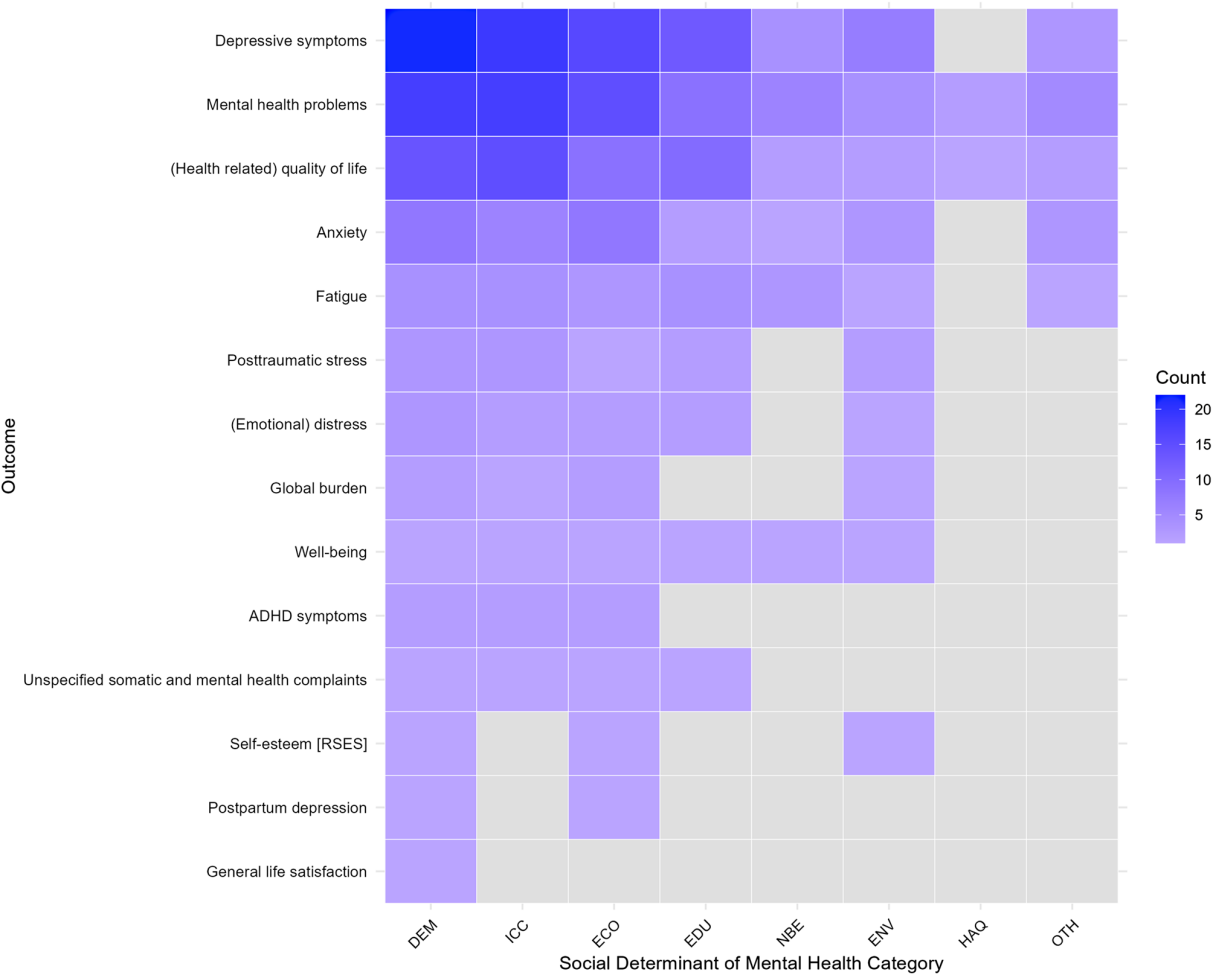
Heatmap visualization of research concentrations between social determinants of health and validated mental health outcomes. *Note*. Similar outcomes measurement instruments were grouped based on the target construct for simpler comparison (for details on grouping, see Supplementary Table S2), while non-validated outcomes (single-item measurements, self-assessments not based on a validated instrument, ICD diagnosis, or visits to physicians or psychiatrists) were excluded. DEM = demographic. ICC = Interpersonal, community, and cultural. ECO = Economic/Economic stability. EDU = education. NBE = Neighborhood and built environment. ENV = environmental events. HAQ = healthcare access and quality. OTH = other health-related components

In addition to the descriptive analysis, Figure 10 (and Supplementary Table S2) shows a heatmap illustrating the frequency of the investigated associations between SDoH domains and MH outcomes. This is based on all measurement instruments recorded and coded in Supplementary Table S2, which were assigned to the SDoH domain categories (DEM, ICC, ECO, EDU, NBE, ENV, HAQ, OTH). The visualization highlights an uneven distribution of research across these relationships: some SDoH are linked to a large number of outcomes, while others are linked to only a few. Specifically, combinations involving DEM, ICC, ECO, and EDU with depression, general MH, and quality of life receive greater attention, whereas combinations involving NBE, ENV, and HAQ, as well as outcomes such as general life satisfaction and self-esteem, are less frequently investigated. Overall, the heatmap reveals a clear pattern in SDoH–outcome relationships, elucidating existing research imbalances.

### Theoretical frameworks, explanatory mechanisms, and the consideration of intersectionality

Regarding theoretical frameworks and explanatory mechanisms, studies applied both general approaches to MH and frameworks focusing on specific subpopulations. Regarding analyses on minority populations (e.g., LGBTQ+ individuals, people with migration history, single parents, or the oldest old), the term ‘vulnerable and marginalized populations’ was frequently used. Concepts assessed in these studies on marginalized groups comprised migration-related stress, acculturation stress, socio-economic disadvantage, loneliness, social exclusion, and stress related to unemployment. A significant number of studies explored risk and protective factors across different life stages. Social capital, resilience, self-efficacy, and social support networks were identified as crucial buffers for MH.

General conceptual models underpin these studies, including the biopsychosocial model [41], social-ecological frameworks [42], or resilience theory, e.g., the risk–resilience model [43]. These models highlight the complex interplay between micro-level factors (e.g., individual lifestyle, coping resources, and biographical stressors) and macro-level influences (e.g., policy, labor market instability, and systemic discrimination). Stress theories, for example, Lazarus’s [44] transactional stress model, Hobfoll’s [45] conservation of resources theory, and the allostatic load model [46] appear frequently in relation to chronic stressors, especially among groups facing migration-related stress, discrimination, and precarious employment. Frameworks such as intersectionality theory [24], social stratification [47], subjective social status, and the capability approach [48] further enrich the analysis of social inequalities in health. Regarding intersectionality, it should be noted that only three studies defined and statistically analyzed intersectionality, while four other studies addressed intersectionality in their discussion. Nevertheless, more than 20 studies used interactions between multiple SDoH that can be considered as intersectionality perspective, even if they were not explicitly labeled as such. Overall, the findings point to a convergence of psychological, social, economic, and more recently environmental SDoH explanatory mechanisms.

## Discussion

This scoping review aimed to provide an overview of the research landscape on SDoH in Germany. The results focused on both MH and MI outcomes. The analysis included 73 studies, with the most frequently examined SDoH domains being demographic factors, interpersonal/community/cultural influences, economic stability, and education. MI was studied more frequently (73.3%) than MH (52.0%) - with some studies addressing both aspects. All studies used self-reported outcome measures and focused on individual-level data, while structural or contextual factors were less systematically captured. Several underlying conceptual frameworks were integrated into the studies, including the biopsychosocial model, socio-ecological perspectives, and resilience theory. The SDOH were often analyzed in the context of a marginalized population. Although the intersectional framework itself was rarely applied explicitly, interactions or moderations, and thus intermediate concepts/models between SDoH and MH were considered.

Concerning our first research question (*Which SDoH are most or least frequently researched?*), the most frequently studied demographic SDoH were sex/gender and age. Many studies relied on binary classifications and used sex and gender interchangeably, which overlooks non-binary or transgender individuals. Age was considered in almost half of the studies. This can be attributed to the fact that age is a readily recorded and non-intrusive variable. While age was often included as a basic demographic characteristic, several studies also conducted age-specific or interaction-based analyses. For example, age was examined in interaction with gender [49–53], physical activity and healthy eating [54], social support [55], and parental status [56], as well as in more complex models including trajectories over time and moderation effects (e.g., involving maternal education) [53]. These findings indicate that age was not only descriptively reported but, in some cases, analytically integrated to explore differential effects across population subgroups. As Germany is an ageing society [30, 31], addressing issues of the oldest old, such as loneliness, is crucial. Yet, the limited attention to other demographic variables highlights the need for standardized, inclusive procedures for collecting and analyzing demographic data, especially regarding gender and diversity in general [57]. In the domain of economic stability, the most frequently assessed construct was SES, typically operationalized through a combination of education, occupation, and household income [51] e.g. [58–60]; this composite approach is widely used in health research due to its ability to capture multidimensional aspects of economic position. However, poverty-related factors, though highly relevant in Germany [34], were rarely examined, despite clear links between material insecurity and MI [17]. In the domain of interpersonal, community, and cultural variables, the most frequently studied variables were ‘marital status’, ‘migration background’, and ‘social support’. However, other indicators (than marital status) for family structure and relations, such as family climate, family conflicts, family constellation, family separation, family situation, family size, family stressors, family structure, family support, family type, etc., maybe even better capturing family structures, were only collected once or twice.

Due to the historical context of so-called “Gastarbeiter” (“guest workers”), late emigrants, and refugees [61], and probably also due to ethical restrictions (e.g., fear of discrimination), German research mostly assesses the history of migration instead of race/ethnicity. Discrimination is increasingly recognized as a key risk factor for MH, particularly in depression and anxiety disorders [19], and some studies applied the minority stress model to examine the impact of stigma and structural discrimination on marginalized populations. On the other hand, the protective factor for SDoH, namely social support, has been examined as a buffer against stress, particularly in ‘high-burden’ populations such as single parents, minority groups, and individuals with a migration background, or the oldest old, through mitigating loneliness. Within the studies, we found that the implementation of social support has varied significantly. Future studies could also explore other personal and community resources more regularly. The domain of education was frequently analyzed through measures of educational attainment, often categorized using years of schooling, or through simplified groupings like ‘low,’ ‘medium,’ and ‘high.’ While the relationship between low education and increased risk for MI is well established, the reviewed studies rarely moved beyond attainment to explore for exemplary structural barriers in the education system.

Overall, our findings suggest that greater emphasis was placed on demographic factors, interpersonal, community, and cultural influences, economic stability, and education, while less attention was given to environmental events, the neighborhood and built environment, healthcare, and other domains. It is worth noting that an increasing number of studies have begun to incorporate self-rated measures, such as the subjective SES, or self-reported health, underscoring a move toward integrating personal perceptions into MH assessments.

Regarding the second research question (*What health outcomes are most examined in terms of MH or MI?*), the analysis revealed an imbalance in the literature, with more studies focusing on MI than on MH outcomes. Notably, studies focused on psychiatric disorders, with depression measurements being the first and anxiety measurements the second most frequently studied MI outcome. The focus on depression in the reviewed literature reflects, on the one hand, the high societal relevance and global disease burden of depression [62]. However, on the other hand, it also reflects a pathology-oriented paradigm in psychological research, whereas ‘positive’ indicators like life satisfaction, resilience, or well-being are less investigated, a gap that the field of positive psychology already seeks to address [63]. The reviewed studies used more measurements summed up in general/unspecified mental/physical health or health-related quality of life, such as general life satisfaction, which is a popular index for well-being [64]. This reliance on rather vague and medically oriented measurements does not capture the holistic concept of MH. Some of the included studies also incorporated measurements from both MI and MH, offering a more comprehensive view, although this may also blur the distinction between deficit-based and strength-based constructs, if not well defined. While most literature favors a dimensional approach of MI, the concept of MI is subject to change. A study examining conceptual changes based on prevalence found that the concept of MI is flexible: as certain symptoms become more prevalent, they are being increasingly viewed as signs of MIs [65]. Given that the WHO’s definition of MH encompasses both the absence of illness and the presence of well-being, studies should (1) provide clear and consistent definitions of MH and MI, (2) critically reflect on conceptual shifts over time, and (3) ensure that their methodological approaches adequately capture both deficit-based and strength-based indicators. The analysis of the relationship between SDoHs and assessed MH/MI outcomes (see Fig. 10) sheds light on focus areas of past research and less-explored fields. Particularly striking seems the concentration of data in the area of overlap between the demographic domain and depression-related outcomes, suggesting a concentration of research in this area. This likely reflects both the high availability of demographic data and the central role of depression as a frequently measured outcome in MH research. However, the prominence of depression should be critically reconsidered, encouraging a broader conceptualization of MH. Additionally, less frequent domains such as neighborhood/built environment, environmental events, and healthcare access and quality, as well as outcomes such as general life satisfaction should receive more attention.

Regarding the third research question (*Which theoretical frameworks and explanatory mechanisms are used and tested to explain the link between SDoH and MH outcomes, and is intersectionality considered?*), we found several different theoretical approaches that have been used to theoretically frame and empirically investigate SDoH. These frameworks emphasize risk factors, but also protective factors for MH, and many studies have acknowledged the relevance of interactions or mediation of factors in explaining MH disparities. Across all domains, results reveal a strong focus on SDoH at the individual level, while systemic-level domains/variables remain understudied. Structural explanatory models, linking health disparities to systemic issues like labor market inequality, structural discrimination, or housing insecurity remain underutilized. Indicators from domains such as healthcare access and quality (e.g., systemic barriers) or neighborhood and built environment (e.g., infrastructure, crime, pollution) could offer valuable insights into the contextual factors influencing MH, but these domains were scarcely represented in the included studies. While the breadth of the SDoH framework allows for a more holistic understanding of social inequalities, its complexity also presents challenges for research, as multidimensional and intersectional approaches are needed to adequately capture the range of influencing factors. Notably, the intersectionality framework was rarely applied.

### Strengths and limitations

With 73 studies included, this review provides a broad overview of the state of research in Germany. Although the national focus might limit generalizability to other countries, our systematic analysis of the full spectrum of SDoH in Germany offers a differentiated picture and supports the identification of context-specific research and knowledge gaps. Different measurement approaches and concepts demonstrate the diversity in the field and encourage reflection on operationalizations. At the same time, this heterogeneity makes it difficult to compare studies. Theoretical references, such as biopsychosocial or socioecological approaches became apparent, indicating increasing trends of conceptually grounded research. However, our classification depended on how explicitly theories and concepts were named in the respective study design. The review reveals key gaps in research, for example, regarding experiences of poverty, discrimination, structural conditions, or positively defined MH. Restricting the review to peer-reviewed studies ensured methodological rigor and consistent reporting standards; however, the exclusion of gray literature (e.g., reports or policy documents) may have resulted in the omission of relevant evidence. In addition, the literature search focused on studies explicitly referring to the term SDoH, which may have excluded relevant studies examining similar determinants without using this terminology. This scoping review focused on mapping methodological and conceptual characteristics of the literature rather than synthesizing the empirical findings of individual studies. As a result, the review does not provide a detailed narrative summary of reported associations between specific SDoH and MH outcomes. A systematic synthesis of these findings would represent an additional valuable direction for future research, as it could further deepen the understanding of how specific SDoHs relate to MH outcomes in the German context.

## Conclusions

This scoping review provides a comprehensive overview of research on the SDs of MH in Germany. The evidence reveals a predominant focus on individual-level determinants while systemic, structural, and environmental factors remain markedly underexplored. MI outcomes, particularly depression and anxiety, dominate the research landscape, whereas positively defined MH indicators such as well-being, resilience, and life satisfaction receive limited attention.

Methodologically, the field is characterized by a reliance on self-reports and heterogeneous instruments, which impede comparability across studies. Moreover, the operationalization of key determinants shows significant gaps: gender is often treated in a binary manner, poverty is often reduced to SES, migration is typically represented by a generic “migration background”, and education is largely confined to formal qualifications without accounting for non-formal education. Contextual dimensions–such as healthcare access, neighborhood resources, and environmental exposures–are rarely incorporated.

To advance the field, future research should adopt more theory-driven, multidimensional, and intersectional approaches that integrate structural and contextual determinants alongside individual factors. Thus, while expanding the range of SDoHs considered is important, particular attention should also be paid to their combined and interacting effects. Current evidence indicates that most studies examine determinants in isolation, thereby limiting the ability to capture their joint and potentially synergistic influence on MH. Importantly, this does not imply that all studies must apply increasingly complex operationalizations. Rather, the level of differentiation should be guided by the research question, theoretical framework, and data availability. At the same time, existing research indicates that overly simplified categorizations (e.g., binary gender or dichotomous migration status) may obscure relevant heterogeneity. In line with recent recommendations (e.g., DiMIS [57]), more nuanced and standardized data collection strategies, such as stepwise approaches to capture migration-related characteristics, can help balance analytical depth with feasibility. Standardized and inclusive data collection procedures, particularly regarding gender and social disadvantage, are essential. By addressing these gaps, research can contribute to a more comprehensive understanding of MH disparities and support evidence-based policies aimed at promoting mental well-being across diverse population groups by addressing systemic, structural, and individual inequalities to ensure the human right to health for all.

## Electronic supplementary material

Below is the link to the electronic supplementary material.

Supplementary Material 1

## Data Availability

The datasets used and/or analysed during the current study are available from the corresponding author on reasonable request.

## Abbreviations

CASMIN: Comparative Analysis of Social Mobility in Industrial Nations
GEDA: German Health Update
GERPS: German Emigration and Remigration Panel Study
ISCED: International Standard Classification of Education
KiGGS: German Health Interview and Examination Survey for Children and Adolescents
MeSH: Medical Subject Headings
MH: Mental Health
MI: Mental Illness
PRISMA-ScR: Preferred Reporting Items for Systematic Reviews and Meta-Analyses extension for Scoping Reviews
SD: Social Determinant
SDoH: Social Determinant of Health
SES: Socio-economic Status
SHIP: Study of Health in Pomerania
WHO: World Health Organization
