# Supplementary material for: Social determinants of mental health in Germany: a systematic scoping review mapping the landscape of researched determinants, outcome measures, and explanatory concepts
Source: Int J Equity Health. 2026 Jun 4;25:145. doi: 10.1186/s12939-026-02877-0 (PMC13242142; doi:10.1186/s12939-026-02877-0)
Supplement: Supplementary file 1 — Supplementary Material 1 [file 12939_2026_2877_MOESM1_ESM.docx]

**Table S1**

*Categorization of Assessed Concepts by Social Determinants of Health Across Included Studies*

| *Study* | *Demographics (DEM)* | *Interpersonal, community, and cultural (ICC)* | *Economic / Economic stability (ECO)* | *Education (EDU)* | *Neighborhood and built environment (NBE)* | *Environmental events (ENV)* | *Healthcare access and quality (HAQ)* | *Other health-related determinants (OTH)* |
| --- | --- | --- | --- | --- | --- | --- | --- | --- |
| Adedeji et al., 2023 | age, gender | social support ("community solidarity"), marital status |  | educational attainment |  |  |  |  |
| Aichberger et al., 2012 | age | relationship status | employment status, poverty risk, socio-economic status (as a combination of education level, employment status and income) | educational attainment |  |  |  |  |
| Barbek et al., 2024 | gender, age | migration background | net household income |  |  |  |  | individual health status (somatic symptom severity) |
| Bartig et al., 2023 | age, gender (only included people whose gender as reported in the population register matched the gender stated on their birth certificate (according to self-report in the  questionnaire)) | migration-related characteristics (duration of residence and residence status, sense of belonging to society in Germany, self-reported experiences of discrimination, German language proficiency) |  | educational attainment |  |  |  |  |
| Bartig et al., 2023 | age, gender (gender assigned according to birth certificate (self-reported)) | household composition, discrimination, migration background ("migrant status") | income | educational attainment |  |  |  |  |
| Bau et al., 2011 | age | social situation, migration background, family situation |  |  |  |  |  | weight and menarche status, dietary patterns and exercise behavior, illness in recent weeks |
| Becker-Gruenig et al., 2016 | gender | migration background | socio-economic status |  | geographical distribution |  |  |  |
| Belau et al., 2021 | age, sex/gender, length of stay since the arrival to Germany | country of origin, residence status according to the German residency law, family separation |  |  |  |  |  |  |
| Beutel et al., 2002 | age | partnership, marital status, religious affiliation | net household income | educational attainment | place of residence (Eastern vs. Western Germany), urbanity |  |  |  |
| Beutel et al., 2004 | age | partnership, religious affiliation | net household income | educational attainment | place of residence (Eastern vs. Western Germany), urbanity |  |  |  |
| Beutel et al., 2017 | age, gender | partnership, children, living alone | socio-economic status |  | place of residence |  |  |  |
| Biddle et al., 2024 | age, gender/sex | asylum status, time since arrival in Germany, country of origin | socioeconomic deprivation | educational attainment | federal state |  |  |  |
| Blume et al., 2024 | sex (based on GEDA), age, duration of residence | household size, social support, discrimination, German language proficiency | household income |  |  | experience of flight or persecution during migration |  |  |
| Bolster et al., 2025 | sex (based on SKKIPPI), age | country of origin, native language, utilization of early childhood services, first time parent, committed relationship, single parenthood, number of adults in household, number of children in household, child with disability/chronic illness, relationship, social support, family stressors | socioeconomic status, welfare recipient status, job, financial situation, neighborhood socio-economic status (proportion of unemployment, proportion of employed welfare recipients, proportion of children under 15 years of age living in poverty) | education (high vs. medium/low) |  |  |  | chronic disease, previous depression, previous anxiety disorder, planned pregnancy |
| Borgmann et al., 2019 | gender, age | social support |  | vocational and academic education | federal state |  |  |  |
| Buchcik et al., 2021 | age, sex/gender | marital status, children, migration background | household income | school education (in years) |  |  |  |  |
| Chae et al., 2022 | age, sex (based on registry data) | marital status, community size, social support | socio-economic status (based on education, occupational status, household income) | academic education |  |  |  |  |
| Cohrdes et al., 2018 | age, sex (based on DEGS1) | partnership status, social support | socio-economic status |  |  |  |  | chronic diseases |
| Eibich et al., 2016 | age, sex (based on Berlin Aging Study II) | share of children, seniors, and welfare recipients in neighborhood, social support |  |  | access to shops, access and distance to public transport, problems with crime, crimes per 100,000 residents | problems with pollution, problems with noise | access and distance to physicians, distance to hospital |  |
| Erhart et al., 2024 | age, gender, sexual orientation | migration background |  | education (classified according to the International Standard Classification of Education) |  |  |  |  |
| Georges et al., 2023 | age, sex (based on GSOEP) | migration background, caregiving, family status |  | educational level (based on International Standard Classification of Education, ISCED) |  |  |  |  |
| Giesebrecht et al., 2024 | age, gender, length of stay in Germany | native language, religion, discrimination (ethnic), discrimination (institutional), in-group identification, social support |  | education (in years) |  |  |  |  |
| Grochtdreis et al., 2024 | age, sex/gender, citizenship | marital/relationship status, religious affiliation, years since migration to Germany, connectedness with country of origin, feeling German, steady relationship (before migration), discrimination, German language skills | employment | school-leaving qualification (secondary general school, secondary school, academic secondary school, and no school-leaving qualification) |  | main reason for migration |  |  |
| Hajek et al., 2016 | age, sex/gender | marital status, living situation |  | educational level |  |  |  |  |
| Hajek et al., 2021 | age, sex/gender | marital status, living situation, size of social network | income poverty, asset poverty | educational level (based on International Standard Classification of Education, ISCED) |  |  |  |  |
| Hajek et al., 2022 | age, sex (based on AgeQualiDe) | marital status |  | education (classified using the Comparative Analysis of Social Mobility in Industrial Nations, CASMIN) |  |  |  |  |
| Hajek et al., 2023 | age, gender/sex | marital status, living situation, size of social network |  |  |  |  |  |  |
| Hajek et al., 2023 | age | family situation, migration background, religions affiliation | employment status | highest school education |  |  |  | having had gender reassignment surgery, chronic diseases, self-reported health, sport activity |
| Hammersen et al., 2016 | age, sex (based on GEDA) | social support | socio-economic status |  | urbanization grade | noise |  |  |
| Hetzel et al., 2016 | age, gender | family status | company size, company orientation |  |  |  |  |  |
| Hoebel et al., 2013 | age, gender | subjective social status | socio-economic status (based on education, income, job position), income, occupational status | education (as a SES core dimension; classified using the Comparative Analysis of Social Mobility in Industrial Nations, CASMIN) |  |  |  |  |
| Hoebel et al., 2017 | age, sex/gender | subjective social status | socio-economic status (based on education, occupational status, household income), occupation, income | educational attainment |  |  |  |  |
| Hollederer et al., 2016 | age, gender, citizenship | living situation/household members | income (monthly equivalent income), deprivation index, work orientation index, job position | education (low, middle, high), student/pupil status | place of residence (Eastern vs. Western Germany) |  |  |  |
| Horsfield et al., 2020 | age, gender | marital status | mean household income | school education (>/=/< 10 years) |  |  |  |  |
| Karl et al., 2024 | age, sex (not specified) |  | employment precariousness |  |  |  |  |  |
| Kirkcaldy et al., 2004 | age, gender |  |  |  |  |  |  |  |
| Kroll et al., 2016 | age, gender | living situation/household members, parenthood | labor force participation, weekly working hours | educational attainment | federal state |  |  |  |
| Kroll et al., 2016 |  |  | unemployment |  |  |  |  |  |
| Kuehne et al., 2015 | age, gender | undocumented migration background |  |  |  |  |  |  |
| Laemmle et al., 2013 |  | immigration background | professional qualification, occupation, net household income | educational attainment | rural-urban differences |  |  |  |
| Limm et al., 2012 | gender/sex, age | migration background, marital/partnership status | duration of unemployment | educational attainment |  | personal migration experience |  | hypertension, smoking habits, level of physical activity |
| Lohaus et al., 2017 | age of the children, children’s sex (based on GROW&TREAT study) | foster vs. biological families |  |  |  |  |  |  |
| Lotty et al., 2015 | age, sex/gender | residence status |  |  | region of origin |  |  |  |
| Lüschen et al., 1997 | age, gender | personal influence among family/friends, power in community, power in politics, subjective social status, work situation (physical strenuous, work stress/noise, problems in social relations at work) | family income, occupation | education (in years) |  |  |  |  |
| Massag et al., 2023 | age, sex (based on DigiHero) |  | fear of worsening of individual financial situation |  |  | fear of war, fear of the exacerbation of consequences of the climate crisis, fear of expansion of military conflict to other countries |  |  |
| Meyrose et al., 2018 | age, gender | family structure (with/without biological parents), migration background, community size, living with biological parents | maternal employment, family income | maternal education (classified using the Comparative Analysis of Social Mobility in Industrial Nations, CASMIN) |  |  |  | maternal mental health |
| Morales et al., 2022 | age, sex(based on SOEP) |  | socio-economic status |  | place of residence (federal state within Germany) |  | depression treatment |  |
| Mugambwa et al., 2023 | age, gender, nationality | migration background/immigration status, country of origin | private economic situation |  | housing status |  | health insurance coverage |  |
| Naeher et al., 2020 | age, gender | marital status, social integration | socio-economic status (based on education, occupational status, household income) | education (classified using the Comparative Analysis of Social Mobility in Industrial Nations, CASMIN) |  |  |  | suicide rates |
| Petrowski et al., 2021 | age, gender |  | income |  |  | pollution |  |  |
| Pfoertner et al., 2022 | gender/sex, age, nationality | marital status, household type | precariousness of employment (based on working poverty, working time arrangements, perceived job insecurity, low social rights), mobility in precariousness of employment |  | region of residence (East vs. West) |  |  |  |
| Pinker et al., 2021 | age | single mother, migration background, social support, experienced social and emotional strains, living situation/household members, subjective social status | employment status before maternity leave, occupational group/job position | maternal educational status (</=/> 10 years, other) | size of current residence, humidity, stains on the wall, smoker in household |  | health insurance, healthcare utilization, doctors' consultation during pregnancy | drinking alcohol around pregnancy, smoking (lifetime, during pregnancy, and since delivery), BMI, diet, history of psychiatric disorders & somatic diseases, birth mode/first child, preterm delivery, child health, breastfeeding, parental stress (index based on personal limitation, parental competence, parent-childhood-bond), physical activity |
| Poethko-Müller et al., 2024 | sex (which sex was entered on your birth certificate at birth), age | relationship status |  | education (classified with the Comparative Analysis of Social Mobility in Industrial Nations, CASMIN) |  |  |  | general health status, chronic diseases, long-/post-covid |
| Rattay et al., 2020 | age, sex/gender | parental status/living with children, marital/relationship status, age of the youngest child | socio-economic status, employment status |  | region of residence |  |  |  |
| Ravens-Sieberer et al., 2009 |  | familiy resources (family climate, family support), social resources (social support from peers and others), school environment (school adjustment, school success, school climate) | socio-economic status (based on education, occupational status, household income) |  |  |  |  |  |
| Ravens-Sieberer et al., 2012 | age, gender |  | socio-economic status | education assessed through Winkler-Index |  |  |  |  |
| Reuter et al., 2023 | age, sex (based on BIBB/BAuA), nationality | country of origin, job demands (six dimensions: ergonomic demands, environmental demands, social support, decision latitude, psychological demands, working time demands) | company characteristics (company size, economic situation of the company, whether company introduced health promotion measures within the past two years, downsizing measures in the past two years), employment relation, weekly working hours, job tenure, economic sector | school education (distinguished between low (<12 years) and high education (≥12 years) | region of residence |  |  |  |
| Rothermund et al., 2003 | age, gender | social resources (number of close friends, perceived social support from friends and relatives) | economic resources (net family income) |  |  |  |  | coping competences, time perspective, health status |
| Santos-Hövener et al., 2019 | gender, age | migration background, parental residence duration | socio-economic status |  |  |  |  |  |
| Schaffrath et al., 2024 | age, gender, residence status | marital status, children | work permit |  |  | post-migration life difficulties, potentially traumatizing events |  |  |
| Scharte et al., 2013 | age, sex, (based on GME), nationality | family type, family size | maternal employment status, maternal income | maternal education |  | environmental factors, noise, traffic load |  |  |
| Schilz et al., 2023 | age, gender/sex, permanent residence permit | time since migration arrival, marital/relationship status, living conditions, social support |  | school education (in years) |  | exposure to potentially traumatic events |  |  |
| Schunck et al., 2015 | age, gender, German citizenship | discrimination, migration background ("migrant status"), marital status | socio-economic status, income | SOEP’s implementation of the International Standard Classification of Education |  |  |  |  |
| Schwager et al., 2020 | gender, age | social integration |  |  |  |  |  |  |
| Schwarz et al., 2007 | age | marital status | socio-economic status (based on education, occupational status, household income) | education (composite indicator of school and vocational education) |  |  |  |  |
| Soeder et al., 2025 | gender/sex, age | migration background, subjective social status | side job, received funding from federal law on support in education | type of university attended, area of study, semester of study | living situation | climate change worry |  |  |
| The BELLA Study Group et al., 2008 | age, gender | single parent or with a step-parent, conflicts in the family, social support, early parenthood, number of older and younger siblings, parental strain, familial resources (family climate, parental support), and social resources (social support, peer competence) | socio-economic status, burden through unemployment of one parent |  |  |  |  | alcohol consumption, unwanted pregnancy, parental health-related quality of life, parental psychiatric symptom, mental disorder or a chronic disease in one parent |
| The BELLA Study Group et al., 2015 | age, gender | family climate, social support | socio-economic status (Winkler-Index: considering education, profession, income) | education assessed through Winkler-Index |  |  |  |  |
| Vonneilich et al., 2011 | age, gender | social integration | socio-economic status (based on education and income) | education (used as SES measures; classified according to the International Standard Classification of Education) |  |  |  |  |
| Waldhauer et al., 2018 | age, gender | migration background | socio-economic status | type of school attended |  |  |  |  |
| Walther et al., 2020 | age, gender, citizenship/nationality | legal/migration status, family constellation in German, household situation |  | education (classified according to the International Standard Classification of Education) |  |  |  |  |
| Wuestner et al., 2019 | age, gender | social support | socio-economic status |  |  |  |  |  |
| Zeier et al., 2024 | gender, age |  |  |  |  | eco-guilt, ecological grief, eco-anxiety |  |  |

*Note*. The classification of the assessed Social Determinants of Health followed a combined inductive and deductive approach. For further details, please refer to the Methods section.

**Table S2**

*Research Concentrations between Social Determinants of Mental Health and Validated Mental Health Outcomes*

| *Validated mental health outcome* | *DEM* | *ICC* | *ECO* | *EDU* | *NBE* | *ENV* | *HAQ* | *OTH* |
| --- | --- | --- | --- | --- | --- | --- | --- | --- |
| **Depressive symptoms** [CES-DC, CIDI-MDD, DIA‐S4, GDS, HADS-D, PHQ-2, PHQ-4, PHQ-8, PHQ-9] | 22 | 19 | 16 | 13 | 4 | 7 | 0 | 3 |
| **Mental health problems** [CBCL, parent-reported SDQ, SDQ, SF-12, SF-36] | 18 | 18 | 15 | 9 | 6 | 4 | 2 | 5 |
| **(Health related) quality of life** [CDC HRQOL-4, EQ-5D, KINDL-R, KIDSCREEN-10, WHOQOL-BREF, ZSL] | 14 | 15 | 9 | 10 | 2 | 2 | 1 | 2 |
| **Anxiety** [GAD-2, GAD-7, SCARED-D, WI-7] | 8 | 6 | 8 | 2 | 1 | 3 | 0 | 3 |
| **Fatigue** [FAS, JSS, MFI-20] | 4 | 4 | 3 | 4 | 3 | 1 | 0 | 1 |
| **Posttraumatic stress** [HTQ, PDS] | 3 | 3 | 1 | 2 | 0 | 2 | 0 | 0 |
| **(Emotional) distress** [GHQ-28, PDI, RHS-13] | 3 | 2 | 2 | 2 | 0 | 1 | 0 | 0 |
| **Global burden** [SCL-90-R, SCL-K9] | 2 | 1 | 2 | 0 | 0 | 1 | 0 | 0 |
| **Well-being** [WHO-5] | 1 | 1 | 1 | 1 | 1 | 1 | 0 | 0 |
| **ADHD symptoms** [CGI-P] | 2 | 2 | 2 | 0 | 0 | 0 | 0 | 0 |
| **Unspecified somatic and mental health complaints** [BL-38] | 1 | 1 | 1 | 1 | 0 | 0 | 0 | 0 |
| **Self-esteem** [RSES] | 1 | 0 | 1 | 0 | 0 | 1 | 0 | 0 |
| **General life satisfaction** [FLZM] | 1 | 0 | 1 | 0 | 0 | 1 | 0 | 0 |
| **Postpartum depression** [EPDS] | 1 | 0 | 1 | 0 | 0 | 0 | 0 | 0 |

*Note*. Similar outcomes measurement intruments were grouped based on the target construct for simpler comparison, while non-validated outcomes (single-item measurements, self-assessments not based on a validated instrument, ICD diagnosis, and visits to physicians or psychiatrists) were excluded. DEM = Demographic. ICC = Interpersonal, community, and cultural. ECO = Economic / Economic stability. EDU = Education. NBE = Neighborhood and built environment. ENV = Environmental events. HAQ = Healthcare access and quality. OTH = Other health-related determinants.
